# The Evolving Demographic and Health Transition in Four Low- and Middle-Income Countries: Evidence from Four Sites in the INDEPTH Network of Longitudinal Health and Demographic Surveillance Systems

**DOI:** 10.1371/journal.pone.0157281

**Published:** 2016-06-15

**Authors:** Ayaga Bawah, Brian Houle, Nurul Alam, Abdur Razzaque, Peter Kim Streatfield, Cornelius Debpuur, Paul Welaga, Abraham Oduro, Abraham Hodgson, Stephen Tollman, Mark Collinson, Kathleen Kahn, Tran Khan Toan, Ho Dang Phuc, Nguyen Thi Kim Chuc, Osman Sankoh, Samuel J. Clark

**Affiliations:** 1 Regional Institute for Population Studies (RIPS), University of Ghana, Legon, Ghana; 2 School of Demography, The Australian National University, Canberra, Australia; 3 MRC/Wits Rural Public Health Transitions Research Unit (Agincourt), School of Public Health, Faculty of Health Sciences, University of the Witwatersrand, Johannesburg, South Africa; 4 CU Population Center, Institute of Behavioral Science, University of Colorado at Boulder, Boulder, Colorado, United States of America; 5 International Centre for Diarrhoeal Diseases Research, Dhaka, Bangladesh; 6 Navrongo Health Research Centre, Navrongo, Ghana; 7 Umeå Centre for Global Health Research, Division of Epidemiology and Global Health, Department of Public Health and Clinical Medicine, Umeå University, Umeå, Sweden; 8 INDEPTH Network, Accra, Ghana; 9 Filabavi Health and Demographic Surveillance Site, Hanoi, Vietnam; 10 Department of Sociology, University of Washington, Seattle, Washington, United States of America; 11 ALPHA Network, London School of Hygiene and Tropical Medicine, London, United Kingdom; 12 Mailman School of Public Health, Columbia University, New York, New York, United States of America; St. Michael's Hospital, CANADA

## Abstract

This paper contributes evidence documenting the continued decline in all-cause mortality and changes in the cause of death distribution over time in four developing country populations in Africa and Asia. We present levels and trends in age-specific mortality (all-cause and cause-specific) from four demographic surveillance sites: Agincourt (South Africa), Navrongo (Ghana) in Africa; Filabavi (Vietnam), Matlab (Bangladesh) in Asia. We model mortality using discrete time event history analysis. This study illustrates how data from INDEPTH Network centers can provide a comparative, longitudinal examination of mortality patterns and the epidemiological transition. Health care systems need to be reconfigured to deal simultaneously with continuing challenges of communicable disease and increasing incidence of non-communicable diseases that require long-term care. In populations with endemic HIV, long-term care of HIV patients on ART will add to the chronic care needs of the community.

## Introduction

The dynamics of health and social transitions in Africa and Asia have created unexpected precedents that give rise to new challenges. Research has demonstrated the uniqueness and complexity of these changes [1, 2]–highlighting the need for robust, prospective data. Such data provide detailed descriptions and more nuanced understandings of transition patterns and variation across settings and time and thereby contribute new perspectives to epidemiological transition theory [3]. Rich longitudinal data also provide the detail and predictive power necessary for informed planning and policy to address health burdens and inequities in lower- and middle-income countries [4].

Sub-Saharan Africa continues to suffer a burden of infectious disease that exceeds the burden from non-communicable disease and injuries. The HIV/AIDS pandemic has led to increased deaths due to infectious illness [5], while the rollout of anti-retroviral therapy (ART) in some countries has improved life expectancy [6, 7]. While countries are at different stages of the epidemiological transition [8], with evolving sex-age and socioeconomic distributions, the interaction of new therapies and improved coverage parallel behavioral and lifestyle change. Together these patterns result in an older population with increased prevalence of HIV/AIDS, and simultaneously, an increasing risk of chronic, non-communicable diseases [9].

In Asia, non-communicable diseases are the leading cause of adult deaths in nearly every country. However, disease burdens vary widely across countries. For example, there is remarkable heterogeneity both within and between India and China [10, 11]. Yet there remains important burdens of tuberculosis and malaria in areas such as India and parts of Southeast Asia [12].

Demographic and mortality changes are interacting with these transitional cause of death profiles to change population structure, with a shift toward proportionally more older persons [9]. Aging populations will add to the burden of non-communicable disease in these countries [13]. Complex social changes are also occurring–such as diet and lifestyle–leading to a shift in risk factors among adults [11].

While regional and global appraisals of trends in disease risk are critical to our comparative understanding [2], a weakness is the lack of small-area outcomes data to inform health systems development–particularly given dynamic demographic, epidemiological, and social change. A data system of high potential is the INDEPTH Network of health and demographic surveillance systems (HDSS) [14, 15], covering over 40 geographically defined rural and urban sites–often in hard to reach, underserved settings. These sites support household- and community-based longitudinal collection of vital events and epi-demographic data, providing a rich research platform for comparative work. HDSSs begin with a baseline census that defines the initial population, usually within a well-defined geographic boundary. Information describing members of the population and the households that they belong to is updated at regular intervals. Members of the population are added through birth or migration into the HDSS area and removed through death or out-migration. Other information is often collected, including marriage and divorce, educational attainment, and immunizations of children. Additionally HDSSs tend to align with local administration boundaries, providing opportunity to inform local planning/evaluation and national policy.

This study is motivated by the fact that there has been little empirical characterization of the demographic and epidemiological transition in the developing countries of Africa and Asia since Omran’s seminal publication in the early 1970s [3]. Yet it is stated that sex and age structure and cause of death patterns are changing in these settings. Many of the publications in the intervening period either use model-based data gathered from health facilities in developing countries, are focused on specific segments of the population, or are based on data from specific countries where cause of death data are available [16–18]. In particular, there is no known work based on empirical, longitudinal population level data across different settings of Africa and Asia.

This paper draws on longitudinal empirical population level data gathered by the INDEPTH Network of field sites to provide new evidence of the evolving demographic and epidemiological transition occurring in Africa and Asia. This study illustrates how data from these sites can provide a comparative, longitudinal examination of mortality patterns and the epidemiological transition. We use pooled data from four HDSS sites and a common statistical framework to investigate mortality trends and their variation over time.

## Method

### Ethical statements

#### Matlab

Matlab HDSS is a subsidiary of the International Center for Diarrhoel Diseases Research, Bangladesh (ICDDR,B) and is guided by the ethics policies of ICDDR,B which are based on the principles of the code of ethics. The Board of Trustees, management, and staff ensures that the affairs of ICDDR,B are conducted honestly and prudently exercising their best care, skill and judgment for the benefit of both research participants and ICDDR,B. Researchers are mandated to act loyally, impartially, and objectively in discharging their official duties with highest standard of professionalism and integrity. Prior to collecting any data, oral consent is sought before proceeding. All members of the Matlab HDSS are informed about the benefits of the research conducted on the backbone of the HDSS and members of the Matlab research team work closely with community leaders to ensure that there is active community participation. Matlab has been in operation for more than 40 years and has, therefore, established rapport with the community and has enjoyed the active support of members of the community and their leadership. Any new research activity that has to be conducted is often communicated to members of the community and research results are periodically shared with communities.

#### Agincourt

The Agincourt health and socio-demographic surveillance system (HDSS) was reviewed and approved by the University of the Witwatersrand Human Research Ethics Committee (Medical) (protocol M110138). Informed consent is obtained for individuals and households at each follow-up visit. Participants provide verbal consent prior to each census interview, and prior to conducting any interview, the fieldworker informs the respondent of the purpose, aims and justification of the study in the first language of the respondent using the fieldworker guidelines, specifically the section on informed consent. Fieldworkers explain to each respondent that they have the option of refusing to participate in the study, and that if s/he declines to participate in the study, no negative consequence will result. Consent has historically been obtained through a verbal consenting process that has continued to be accepted by ethics committees and is in line with normal practice of all INDEPTH (www.indepth-network.org) HDSS sites. Furthermore, community consent is obtained through frequent meetings with community leaders.

#### Navrongo

The Navrongo Health Research Centre (NHRC), the institution that operates the Navrongo HDSS, has an Institutional Review Board (IRB) which was established in February 2002 as an independent regulatory body to review, evaluate and decide on the ethical merits of the NHRC research protocols. The Board ensures that research protocols involving human subjects meets the required ethical standards and it ensures and guarantees the rights, dignity, safety and protection of all individuals and communities who participate in NHRC research activities. NHRC’s IRB’s registration number is FWA00000250 and the identifier is IRB00000802. For the operations of the HDSS, individual oral consent is sought before data are collected from the population. Members are informed about their obligations, that they are not obliged to provide information and that they have the right to refuse to participate in the research without suffering any adverse consequences. In addition, respondents are informed about the benefits and risks for participation. In terms of community involvement, the researchers undertake community sensitization from time to time. Finally, the IRB ensures that there is regular monitoring to ensure compliance with laid down protocols. The operations of the Board are guided by international guidelines and principles.

#### Filabavi

As a unit of Vietnam's Hanoi Medical University, all data collection and research, including the Filabavi health and demographic surveillance system, are guided by the Hanoi medical university code of ethics and approval. Informed consent procedures have been established and these have been in use within the HDSS since 1999 when the HDSS was established. Prior to the conduct of each interview, the research team would normally explain the purpose of the data collection to every respondent and verbal informed consent received. There is also active community participation through sensitization campaigns in the Bavi district where the Filabavi HDSS is operating. In addition to the Hanoi Medical University Ethics Committee, the research Ethics Committee at Umeå University (Sweden), a collaborating institution of the Filabavi HDSS, reviewed and provided approval for the FilaBavi household surveillance system, including data collection on vital statistics.

### Data

Data come from four HDSSs: two in Asia (Matlab, Filabavi) and two in Africa (Navrongo, Agincourt). Each site conducts longitudinal population follow-ups (after an initial census) at regular intervals of the entire population under surveillance in a defined geographic area. Data available for these analyses include sex, age, and survival status (and cause of death for those who died) for each individual observed. Cause of death information comes from physician coded verbal autopsy interviews: when a death is identified trained fieldworkers conduct a verbal autopsy interview with the closest living relative. Physicians then independently review the interview and assign a probable cause of death. If physicians agree on a particular cause of death then it is identified as the cause of death. If they disagree another set of physicians independently review the same information and if there is agreement, the agreed cause is accepted as the probable cause of death; if not (i.e., if there is still disagreement), such deaths are categorized as Indeterminate or ‘ill-defined’. Indeterminate causes of death are excluded from our analysis.

We briefly describe each HDSS below. The Matlab HDSS site is situated in Matlab Upazila (sub-district) under the Chandpur District in Bangladesh, about 55 km southeast of the capital, Dhaka. Established in 1966, fieldworkers visit each household every two months to update vital events. The current total population is about 225,000 people in 142 villages [19]. Cause of death information is not available for the more recent period due to delays in physician review of the verbal autopsy interviews. For Matlab, data are available for all-cause and cause-specific mortality from 1987–2006.

The Agincourt HDSS is located in the Agincourt area of the Bushbuckridge Local Municipality, Ehlanzeni District, Mpumalanga Province of South Africa. Established in 1992, fieldworkers visit each household yearly to update vital events. The current total population is 87,040 people living in 14,382 households and 26 villages [20]. For Agincourt, data are available for all-cause mortality from 1994–2009, and for cause-specific mortality from 1994–2004.

The Navrongo HDSS covers the entire Kassena-Nankana Municipal and West districts (formerly one district) of the Upper East region of Ghana. Since 1993 fieldworkers visit each household quarterly to update vital events. The current total population is 152,000, over a third of which is younger than fifteen years [21]. For Navrongo, data are available for all-cause mortality from 1995–2007 and for cause-specific mortality from 1995–2004.

The Filabavi HDSS is situated in Bavi district, a rural district of Hanoi Province in northern Viet Nam. Established in 1999, follow-up household surveys occur every three months to update vital events. The current total population is 52,000 in 11,089 households [22]. For Filabavi, data are available for all-cause mortality from 1999–2007, and for cause-specific mortality from 2004–2007.

### Analyses

We model mortality using discrete time event history analysis (DTEHA) [23]. We organize the data into person-years, where each person is at risk of dying for each year they are observed (up to and including death). The person-year data include one observation for each observed person-year lived by an individual (i.e., the sample size is observed person-years over the study period). This approach can include time constant and time varying covariates which are defined at the beginning of each person-year. DTEHA provides a multivariate regression-based statistical framework capable of investigating mortality trends by sex and age, their variation over time, as well as the relationships among model-based predictions that describe the epidemiological transition occurring at each site.

#### All-Cause Mortality

We first describe overall mortality patterns over time for each site. We use site-stratified, logistic regression models to estimate the all-cause annual probability of dying by sex, age, and time. We test for all two- and three-way interactions of these predictors by site using nested likelihood ratio tests.

#### Cause-Specific Mortality

We next describe changes in the distribution of deaths by causes over time for each site. We use site-stratified, multinomial logistic regression models to estimate the cause-specific annual probability of dying by sex, age, and time. We test for all two-way interactions of these predictors by site using nested likelihood ratio tests. No three-way interactions were tested. We categorize causes of death, using standard ICD-10 codes, based on information from verbal autopsies conducted in each site. The grouped-cause categories we use are similar to those suggested by the Global Burden of Disease Study [16], with exceptions for commonly occurring, regionally-specific causes of death at two sites–HIV/TB and malaria–which we include as separate cause groups where they occur.

- *Matlab and Filabavi*: ‘communicable diseases’ (including diarrhea, malaria, malnutrition, maternal, other infectious diseases, perinatal, respiratory, and HIV); ‘non-communicable diseases’ (including cancer, cardiovascular, congenital, diabetes, epilepsy, female genital cancer, kidney, liver, and upper GI bleeds); and ‘injuries’ (including accidental injuries, assault, other external causes, suicide, and transport-related).
- *Agincourt*: ‘HIV/TB’; ‘other communicable diseases excluding HIV/TB’ (see above); ‘non-communicable diseases’ (see above); and ‘injuries’ (see above).
- *Navrongo*: ‘Malaria’; ‘other communicable diseases excluding Malaria’ (see above); ‘non-communicable diseases’ (see above); and ‘injuries’ (see above).

#### Epidemiological Transition

We consider two models of the epidemiological transition. Each model excludes Filabavi due to low variation in cause-specific mortality over time.

In the first model we investigate the relationship between the level of all-cause mortality and the probability dying from a cause in one of the broad cause groups. We operationalize this at the individual, person-year level using site-stratified multinomial logistic regression models that relate the probability of dying from a cause in each of the cause groups to the level of all cause mortality. For each site, the all-cause mortality level predictor is derived from the ‘all-cause model’ (above) as the log of the predicted all-cause annual probability of dying averaged over the multi-year historical periods defined in the investigation. For this model we consider the same three cause groups in all sites: communicable diseases, non-communicable diseases, and injuries.

The second model investigates the relationship between all-cause mortality level and the distribution of deaths by cause, using the broad cause groups. Because the aim is to relate the distribution of deaths by cause to the overall level of mortality, this model is operationalized at the site, or community, level rather than the individual level. This model estimates the log ratio of the proportion of deaths in either cause group ‘non-communicable’ or ‘injuries’ relative to the proportion of deaths in the ‘communicable’ group by year. As before, this model uses the site-level, all-cause mortality level predictor, described just above for the first model. Predictions from this model show the direction and magnitude of changes in the distribution of deaths by cause as the level of overall mortality changes. We estimate this model using *seemingly unrelated regression* following the approach employed by Salomon and Murray [17].

## Results

**Fig 1:**
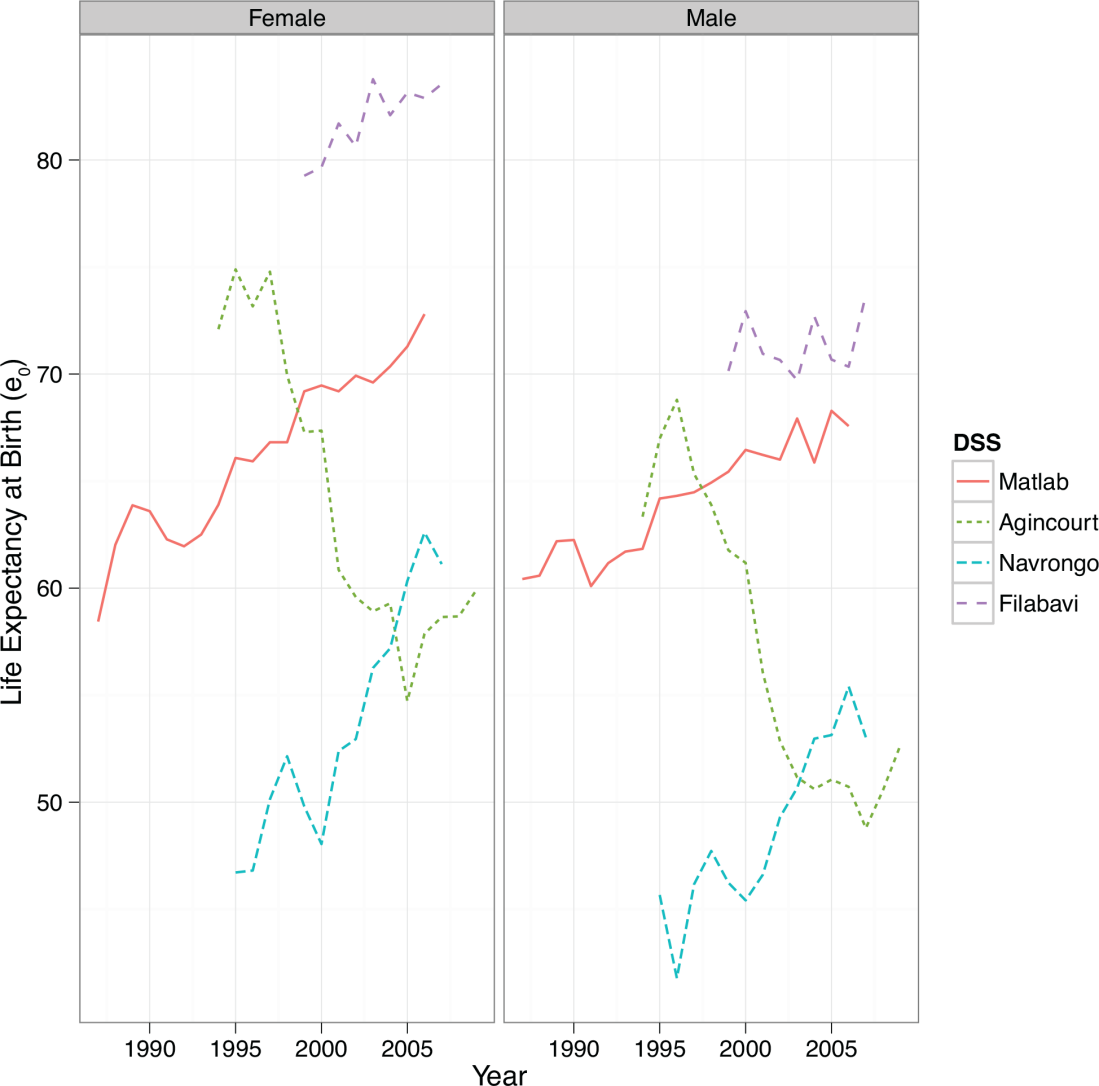
Life expectancy at birth by time and site.

Fig 1 shows life expectancy at birth (*e*_0_) for each site over time. Life expectancy increased for both Matlab and Navrongo, though more rapidly in Navrongo. Filabavi showed little variation in life expectancy at birth over time. In Agincourt life expectancy rapidly declined over time.

**Fig 2:**
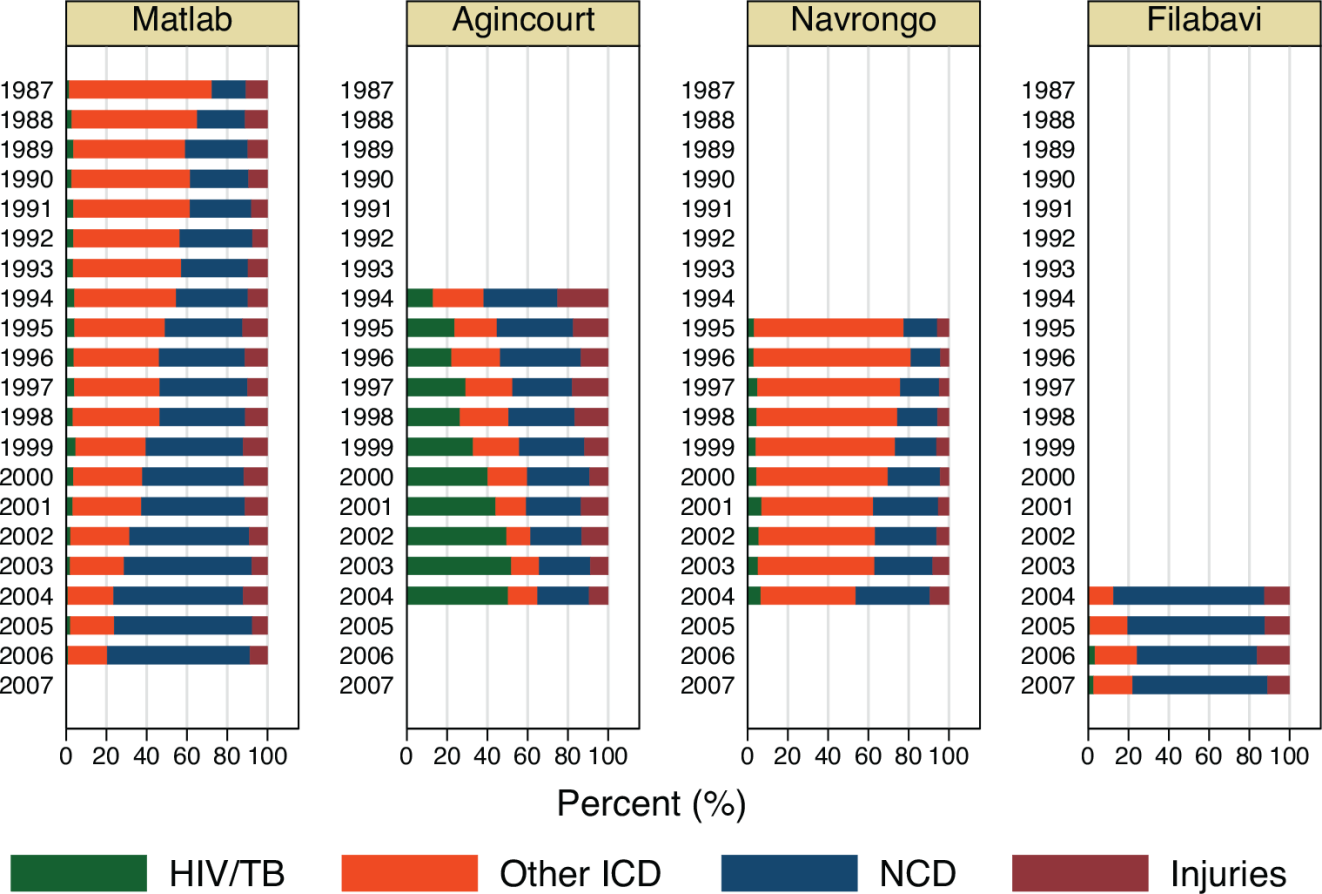
Stacked bar chart of causes of death by time and site.

Fig 2 shows the summed proportion of each major cause of death over time for each HDSS site. In Matlab, deaths due to communicable diseases steadily declined over time, while the proportion of deaths due to noncommunicable diseases increased. In Agincourt, deaths due to HIV/TB steadily increased over time, while noncommunicable disease deaths decreased but remained an important cause of death. In Navrongo, communicable disease deaths declined while the proportion of deaths due to noncommunicable diseases increased. Filabavi showed little systematic change over time, but the major proportion of deaths were due to noncommunicable diseases.

### All-cause mortality

We begin by modeling the relationship between the probability of dying due to any cause by sex, age, and time for each site to describe overall changes in mortality levels. For Matlab, interactions between sex and age (p<0.001), sex and time (p*<*0.001), and age and time (p*<*0.001) improved model fit and were included. Including a three-way interaction of sex, age, and time did not improve model fit and was not included (p = 0.245). For Agincourt, interactions between sex and age (p*<*0.001), and age and time (p*<*0.001) improved model fit and were included. Including a three-way interaction of sex, age, and time improved model fit and was included (p = 0.005). We therefore included an interaction between sex and time (p = 0.691) for model interpretation. For Navrongo, interactions between sex and age (p*<*0.001), sex and time (p*<*0.001), and age and time (p*<*0.001) improved model fit and were included. Including a three-way interaction of sex, age, and time improved model fit and was included (p = 0.007). For Filabavi, interactions between sex and age (p*<*0.001) improved model fit and were included. See S1–S4 Tables for estimation results.

S1 Fig presents the predicted probability of dying from any cause (per 1,000) on the log scale by sex, age and time for each site. Matlab showed a steady decline in the probability of dying over time. Agincourt showed an increase since 2000, particularly for ages 20–50. Navrongo showed a decrease in the probability of dying since 2005, particularly for ages 5–20. Filabavi showed no systematic change in the probability of dying over time.

### Cause-specific mortality

We next modeled the relationship between the probability of dying due to four different cause groups by sex, age, and time for each site to describe changes in cause-specific mortality patterns. For Matlab, interactions between sex and age (p<0.001), sex and time (p<0.001), and age and time (p<0.001) improved model fit and were included. For Agincourt, an interaction between sex and age (p*<*0.001) improved model fit and was included. For Navrongo, interactions between sex and age (p*<*0.001), and age and time (p*<*0.001) improved model fit and were included. For Filabavi, no interactions improved model fit. See S5–S8 Tables for estimation results.

S2 Fig shows the predicted probability of dying due to various causes over time, age, and sex in Matlab. The probability of dying due to communicable diseases declined over time, while the probability of dying due to noncommunicable diseases increased over time—particularly after ages 60+. Injuries showed no systematic pattern over time.

S3 Fig presents the predicted probability of dying due to various causes over time, age, and sex in Agincourt. The probability of dying due to HIV/TB increased substantially since 2000 for ages 20–60. There was relatively little change in the probability of dying due to other communicable diseases, noncommunicable disease, or injuries over time.

S4 Fig presents the predicted probability of dying due to various causes over time, age, and sex in Navrongo. The probability of dying due to Malaria decreased since 2000 for those under age 10 and over age 60. The probability of dying due to other communicable diseases also decreased since 2000 for those over age 40. The probability of dying due to noncommunicable disease and injuries showed little systematic variation over time.

S5 Fig presents the predicted probability of dying due to various causes over time, age, and sex in Filabavi–due to limited data only one time period is shown. The probability of dying from communicable diseases was relatively low across most ages, while the probability of dying due from noncommunicable disease steadily increased into older ages. The probability of dying due to injuries was similar to the other sites, with no evident change over time.

### Epidemiological transition

**Fig 3:**
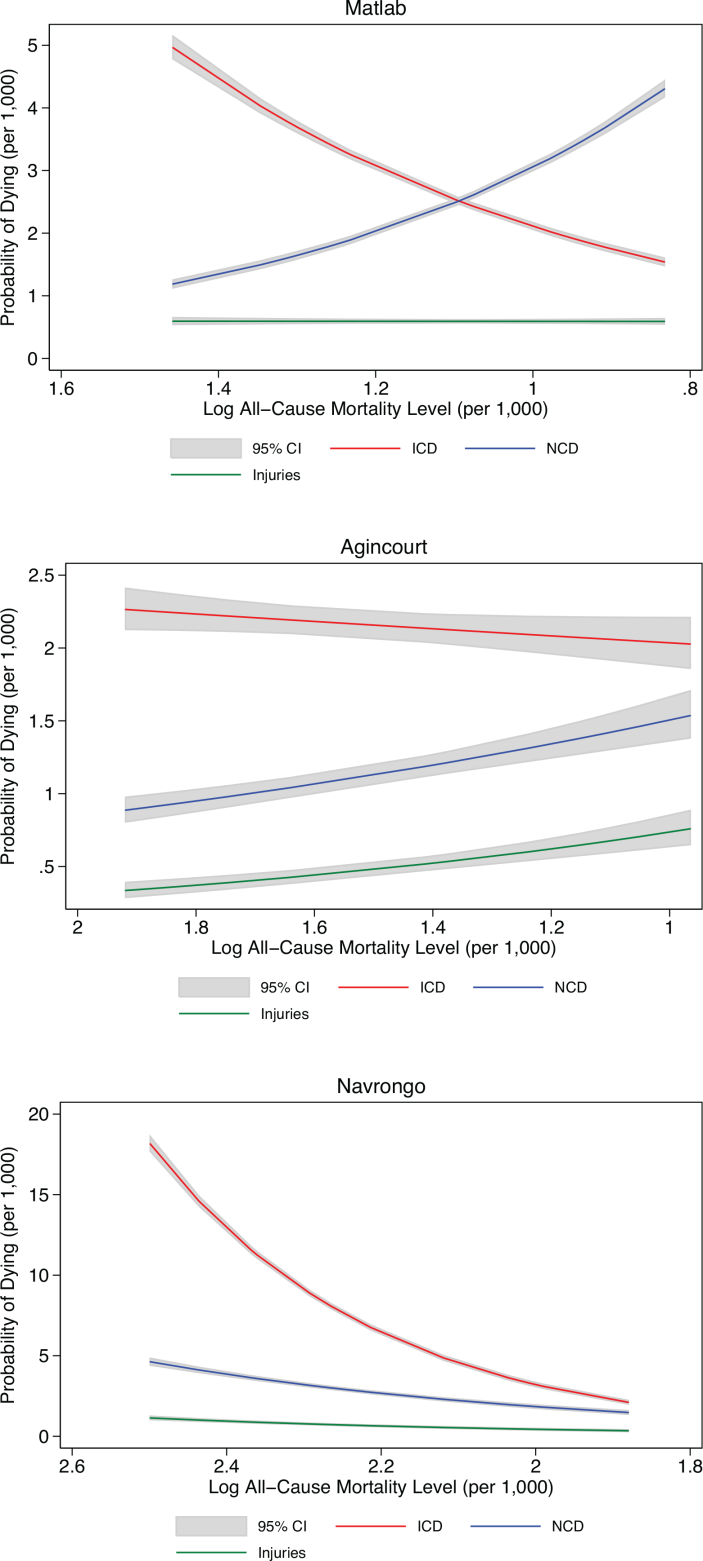
Changes in predicted probability of dying due to cause of death by site and time: individual. X-axis is reversed to show time-trend across all sites except Agincourt, where mortality has been increasing over time.

We next modeled the relationship between changes in distribution of deaths by cause as a function of overall mortality levels at each site, at both the individual-level and site aggregate. At the individual-level, Fig 3 shows the predicted probability of dying from different causes of death in relation to predicted all-cause mortality (see ‘All-cause mortality‘ above) by site (see S9 Table for estimation results). The x-axis is reversed (the all-cause mortality level) to show the pattern over time for all sites (except Agincourt where mortality has been increasing). In Matlab, the probability of dying due to communicable diseases declined as all-cause mortality declined, while the probability of dying due to noncommunicable disease concurrently increased as all-cause mortality declined. In Agincourt, as all-cause mortality increased the probability of dying due to noncommunicable disease and injuries declined while the probability of dying due to HIV/TB remained high. In Navrongo, as all-cause mortality declined the probability of dying due to all three causes also declined.

**Fig 4:**
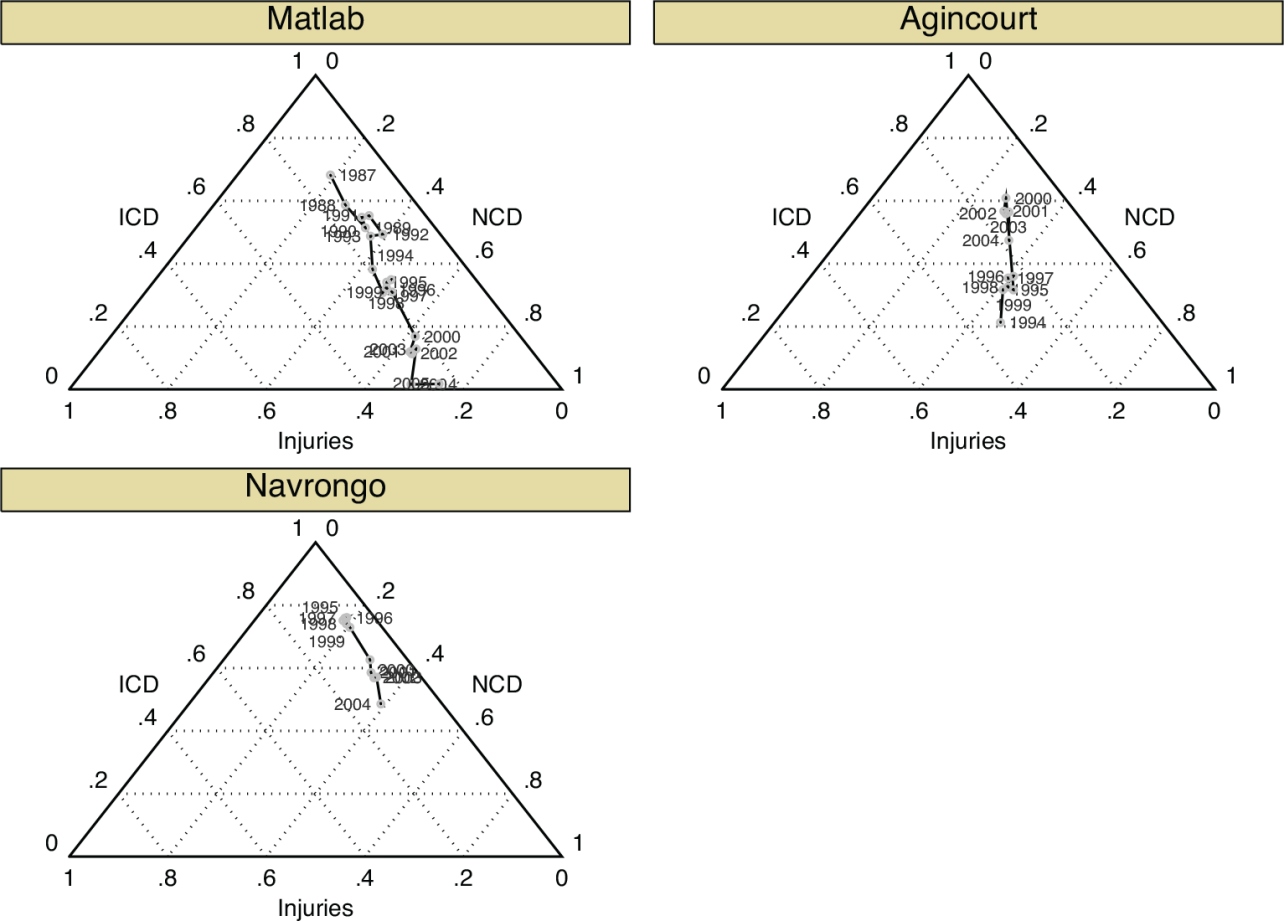
Changes in cause of death distribution by site and time: site. The closer a point is to one of the vertices, the greater the proportion of deaths due to that cause. For instance, in Matlab in 1987, the estimated proportions would be: 68% communicable diseases (ICD), 19% noncommunicable diseases (NCD), and 13% Injuries.

At the aggregate, site-level, Fig 4 shows the fraction of deaths due to various causes over time as a function of all-cause mortality levels (see S10 Table for estimation results)–these predictions show the direction and magnitude of changes in the distribution of deaths by cause as overall mortality levels change. The nearer a point is to one of the vertexes, the greater the proportion of deaths due to that cause. For example, at Matlab in 1987, 68% of deaths were due to communicable diseases, 19% noncommunicable diseases, and 13% due to injuries. For Matlab, the proportion of deaths due to communicable diseases steadily declined, with a resulting increase in the proportion of deaths due to noncommunicable disease–all as the overall level of mortality fell steadily. For Agincourt, the proportion of deaths due to communicable diseases (including HIV/TB) increased over time. In Navrongo, the proportion of deaths due to communicable diseases rapidly declined.

## Discussion

We describe the dynamic health transitions occurring in four HDSSs in Africa and Asia. Agincourt is experiencing a ‘counter’ transition, with increasing overall mortality. The transition is also prolonged [3], with change incomplete and different stages spanned simultaneously. There are continuing high levels of deaths due to communicable disease, largely due to HIV/AIDS and TB. Concurrently, there is a slowly emerging noncommunicable disease burden. This represents a unique form of the epidemiological transition where there is a double burden of noncommunicable diseases in the face of continuing persistence of infectious diseases [24, 25]. In comparison, Matlab is experiencing a classic transition [3] with declines in overall mortality, a reduction in the proportion of deaths due to communicable causes, and a resulting increase in the burden of noncommunicable disease. Navrongo is experiencing a delayed transition [3], largely due to reductions in mortality due to malaria. The disease burden has yet to shift to noncommunicable disease. Finally, Filabavi shows relatively little systematic variation over a short period of observation.

The pattern of changes observed in both demographic trends and cause of death distributions suggests that the demographic and epidemiological transitions are underway in these low and middle-income settings. However, unlike the classical transitions described variously [3, 26, 27], what is happening in these settings represents a mix of gains in medical technology [26] and socioeconomic improvements [27] that have simultaneously led to reductions in communicable diseases and increases in non-communicable diseases, but also a failure of the health systems to respond to and deal with the emerging threat of new/recurring infectious diseases. No doubt these countries have benefited from improvements in vaccinations and modern treatments of common diseases that most likely contributed to the observed decline in communicable diseases, except HIV/TB.

In addition to the improvements in medical and public health technologies, improvements in the socioeconomic conditions in many of the countries of Asia and Africa may help to explain the health and demographic transitions now underway. There has been well-documented, sustained economic growth in Asia over the past few decades and recent reports from the World Bank and other international organizations suggest that Africa is also making impressive strides in terms economic growth, with an annual average growth rate of 4.1% during recent times [28]. These improvements in social and economic conditions, coupled with innovations in medicine and public health interventions, have led to increases in life expectancy and altered dietary patterns, resulting in increases in non-communicable diseases.

However, the case of Agincourt, South Africa is peculiar because of the devastating effect of HIV/AIDS, which until recently (~2007) was not adequately treated with antiretroviral therapy [18]. Mortality resulting from external causes–mainly accidents and injuries–has also emerged recently as a public health priority in developing world settings, and our results support this. Injury mortality in these countries can often be attributed to failure of governments to respond to the needs of rapidly growing populations with a large proportion of young people and with large socioeconomic inequalities resulting in increases in violent crime and rapid motorization of the more affluent segment of the population.

Taken individually, it is important to note that while both Agincourt and Navrongo are African settings, they differ markedly in terms of their wider contexts. Agincourt’s economy historically had better prospects because of the wider economic growth of South Africa compared to Navrongo where the economic conditions were extremely poor and economic growth very slow. Moreover at the onset of the transition, Agincourt’s fertility and mortality indicators were much lower than those of Navrongo. The onset of HIV/AIDS epidemic in South Africa reversed many of the historical improvements in both fertility and mortality. On the other hand, the Matlab site in Bangladesh benefited enormously from long and sustained donor investments in health and social programs, as reflected in the impressive declines in both fertility and mortality. Finally, the Filabavi site in Vietnam started off with very low levels of both fertility and mortality, likely a reflection of that country’s superior public health system.

Ongoing epidemiological transitions have implications for public health and social policy. Within the context of continuing and persistent communicable diseases, health care systems will need to be reconfigured to deal simultaneously with continuing challenges of infectious disease and increasing incidence of non-communicable diseases that require long-term care [18]. In populations with endemic HIV, the long-term care of HIV patients on ART will be added to the growing chronic care needs of the community resulting from noncommunicable diseases.

This study has limitations. First, we analyzed a limited number of sites located in specific geographic areas; future research could include additional sites. Second, cause of death information derived from verbal autopsy is limited by inconsistencies in physician coding over time and across sites. The cause of death data that was submitted to INDEPTH for this work was based on physician coding since that was how cause of death was determined over the periods covered by our analysis. Future work could request verbal autopsy information from sites that would allow machine-based, automated methods for coding verbal autopsy deaths to produce comparable cause assignments across sites and time, but that is not possible for the current research because the underlying verbal autopsy indicator data were not provided by the sites. Third, we exclude a small number of deaths with indeterminate causes. Our general preference is to not construct data using multiple imputation techniques unless the missing data generate empty cells in the analysis, which is not the case here. Fourth, our analysis included a limited number of variables and lacks data on the most recent time periods. At the time when data were submitted by the sites for this work, the most recent cause of death information available was through 2007. Fifth, other aspects of the burden of disease such as morbidity and disability are important to understand the epidemiological profiles of these sites. Data were not available to describe either morbidity or disability. Finally and again because of a lack of data, we cannot include a range of other important social and economic factors that may differentiate and help explain transition trajectories.

The limitations notwithstanding, this study leveraged longitudinal, individual-level data and demonstrated that we can summarize health transition trajectories in a unified, statistical framework with comparable data across surveillance sites. Mortality data from rural areas in low- and middle-income countries is particularly lacking–HDSS data can form an important bridge in expanding our understanding of regional and national epidemiological change. Future work could include additional sites and incorporate variables at multiple levels (e.g., from individual to household, national, and regional). In particular, including social and economic factors from each site could lead to a greater understanding of the underlying causes, timing of the onset, pace, and pattern of the transitions and their variation across space and time. This approach could bring further refinement, extension, and new perspectives to epidemiological transition theory. Further with many more observations (sites and observed time), analyses like this could form the basis for a new set of model life tables for developing countries that describe general patterns of sex-age-specific mortality differentiated by geography, epidemiological epoch and important epidemic disease characteristics such as HIV prevalence and antiretroviral therapy coverage. Finally, developing a stronger evidence base can yield the detail and predictive power that is critical for today’s policymakers and planners to target health burdens and inequities.

## Supporting Information

S1 Fig Site-specific predicted probability of dying by age and time. (DOCX)

S2 Fig Site-specific predicted probability of dying by cause, age, and time: Matlab. (DOCX)

S3 Fig Site-specific predicted probability of dying by cause, age, and time: Agincourt. (DOCX)

S4 Fig Site-specific predicted probability of dying by cause, age, and time: Navrongo. (DOCX)

S5 Fig Site-specific predicted probability of dying by cause, age, and time: Filabavi. (DOCX)

S1 Table Logistic regression of all-cause mortality, Matlab, Bangladesh, 1987–2006 (N = 4,120,472 person years). (DOCX)

S2 Table Logistic regression of all-cause mortality, Agincourt, South Africa, 1994–2009 (N = 1,045,993 person years). (DOCX)

S3 Table Logistic regression of all-cause mortality, Navrongo, Ghana, 1995–2007 (N = 1,600,536 person years). (DOCX)

S4 Table Logistic regression of all-cause mortality, Filabavi, Vietnam, 1999–2007 (N = 425,225 person years). (DOCX)

S5 Table Multinomial logistic regression of cause-specific mortality, Matlab, Bangladesh, 1987–2006 (N = 4,111,333 person years). (DOCX)

S6 Table Multinomial logistic regression of cause-specific mortality, Agincourt, South Africa, 1994–2004 (N = 712,392 person years). (DOCX)

S7 Table Multinomial logistic regression of cause-specific mortality, Navrongo, Ghana, 1995–2004 (N = 1,215,411 person years). (DOCX)

S8 Table Multinomial logistic regression of cause-specific mortality, Filabavi, Vietnam, 2004–2007 (N = 144,713 person years). (DOCX)

S9 Table Multinomial logistic model of all-cause mortality on cause-specific mortality at the individual level, by DSS site. (DOCX)

S10 Table Seemingly unrelated regression model of all-cause mortality on cause-specific mortality at the site-level. (DOCX)
