# Supplementary material for: The Evolving Demographic and Health Transition in Four Low- and Middle-Income Countries: Evidence from Four Sites in the INDEPTH Network of Longitudinal Health and Demographic Surveillance Systems
Source: PLoS One. 2016 Jun 15;11(6):e0157281. doi: 10.1371/journal.pone.0157281 (PMC4909223; doi:10.1371/journal.pone.0157281)
Supplement: S1 Fig — (DOCX) [file pone.0157281.s001.docx]

**Figure S1: Site-specific predicted probability of dying by age and time.**
