# Supplementary material for: The Evolving Demographic and Health Transition in Four Low- and Middle-Income Countries: Evidence from Four Sites in the INDEPTH Network of Longitudinal Health and Demographic Surveillance Systems
Source: PLoS One. 2016 Jun 15;11(6):e0157281. doi: 10.1371/journal.pone.0157281 (PMC4909223; doi:10.1371/journal.pone.0157281)
Supplement: S1 Table — (DOCX) [file pone.0157281.s006.docx]

**Table S1. Logistic regression of all-cause mortality, Matlab, Bangladesh, 1987–2006 (N = 4,120,472 person years).**

| Variable | Odds Ratio | 95% CI | p-value |
| --- | --- | --- | --- |
| *Sex* |  |  |  |
| Male | 0.959 | [0.911, 1.010] | 0.112 |
| *5-Year Age Groups* |  |  |  |
| 0–4 | 1.000 | – | – |
| 5–9 | 0.068 | [0.058, 0.079] | < 0.001 |
| 10–14 | 0.029 | [0.023, 0.037] | < 0.001 |
| 15–19 | 0.046 | [0.037, 0.057] | < 0.001 |
| 20–24 | 0.065 | [0.053, 0.079] | < 0.001 |
| 25–29 | 0.086 | [0.072, 0.103] | < 0.001 |
| 30–34 | 0.077 | [0.064, 0.094] | < 0.001 |
| 35–39 | 0.099 | [0.081, 0.120] | < 0.001 |
| 40–44 | 0.141 | [0.118, 0.168] | < 0.001 |
| 45–49 | 0.168 | [0.142, 0.197] | < 0.001 |
| 50–54 | 0.294 | [0.258, 0.335] | < 0.001 |
| 55–59 | 0.419 | [0.373, 0.472] | < 0.001 |
| 60–64 | 0.827 | [0.748, 0.915] | < 0.001 |
| 65–69 | 1.59 | [1.450, 1.744] | < 0.001 |
| 70–74 | 2.764 | [2.523, 3.029] | < 0.001 |
| 75–79 | 4.049 | [3.664, 4.476] | < 0.001 |
| 80–84 | 5.842 | [5.179, 6.590] | < 0.001 |
| 85+ | 8.989 | [7.851, 10.293] | < 0.001 |
| *Time Period* |  |  |  |
| 1985–1989 | 1.197 | [1.125, 1.273] | < 0.001 |
| 1990–1994 | 1.000 | – | – |
| 1995–1999 | 0.73 | [0.686, 0.777] | < 0.001 |
| 2000–2004 | 0.538 | [0.504, 0.575] | < 0.001 |
| 2005–2009 | 0.385 | [0.347, 0.427] | < 0.001 |
| *Interactions between Sex and Age* |  |  |  |
| Male ***X*** age 5–9 | 1.079 | [0.929, 1.253] | 0.317 |
| Male ***X*** age 10–14 | 1.025 | [0.832, 1.264] | 0.814 |
| Male ***X*** age 15–19 | 0.801 | [0.665, 0.967] | 0.021 |
| Male ***X*** age 20–24 | 0.738 | [0.613, 0.888] | 0.001 |
| Male ***X*** age 25–29 | 0.951 | [0.788, 1.147] | 0.598 |
| Male ***X*** age 30–34 | 1.03 | [0.854, 1.243] | 0.757 |
| Male ***X*** age 35–39 | 1.287 | [1.080, 1.533] | 0.005 |
| Male ***X*** age 40–44 | 1.592 | [1.361, 1.862] | < 0.001 |
| Male ***X*** age 45–49 | 1.656 | [1.429, 1.918] | < 0.001 |
| Male ***X*** age 50–54 | 1.837 | [1.625, 2.078] | < 0.001 |
| Male ***X*** age 55–59 | 1.845 | [1.658, 2.053] | < 0.001 |
| Male ***X*** age 60–64 | 1.527 | [1.396, 1.671] | < 0.001 |
| Male ***X*** age 65–69 | 1.256 | [1.156, 1.364] | < 0.001 |
| Male ***X*** age 70–74 | 1.044 | [0.962, 1.134] | 0.303 |
| Male ***X*** age 75–79 | 0.99 | [0.907, 1.081] | 0.823 |
| Male ***X*** age 80–84 | 0.914 | [0.824, 1.014] | 0.089 |
| Male ***X*** age 85+ | 0.942 | [0.838, 1.058] | 0.311 |
| *Interactions between Sex and Time* |  |  |  |
| Male ***X*** 1985–1989 | 0.942 | [0.880, 1.008] | 0.086 |
| Male ***X*** 2000–2004 | 1.06 | [0.996, 1.127] | 0.067 |
| Male ***X*** 2005–2009 | 1.121 | [1.053, 1.194] | < 0.001 |
| *Interactions between Age and Time* |  |  |  |
| 1985–1989 ***X*** age 5–9 | 1.012 | [0.824, 1.243] | 0.908 |
| 1985–1989 ***X*** age 10–14 | 0.895 | [0.637, 1.259] | 0.525 |
| 1985–1989 ***X*** age 15–19 | 1.087 | [0.807, 1.463] | 0.584 |
| 1985–1989 ***X*** age 20–24 | 1.225 | [0.939, 1.598] | 0.134 |
| 1985–1989 ***X*** age 25–29 | 0.872 | [0.674, 1.130] | 0.301 |
| 1985–1989 ***X*** age 30–34 | 0.891 | [0.661, 1.203] | 0.452 |
| 1985–1989 ***X*** age 35–39 | 0.705 | [0.516, 0.964] | 0.029 |
| 1985–1989 ***X*** age 40–44 | 0.749 | [0.576, 0.974] | 0.031 |
| 1985–1989 ***X*** age 45–49 | 0.909 | [0.725, 1.141] | 0.412 |
| 1985–1989 ***X*** age 50–54 | 0.911 | [0.764, 1.086] | 0.299 |
| 1985–1989 ***X*** age 55–59 | 0.985 | [0.841, 1.153] | 0.849 |
| 1985–1989 ***X*** age 60–64 | 0.924 | [0.801, 1.066] | 0.277 |
| 1985–1989 ***X*** age 65–69 | 0.785 | [0.685, 0.900] | 0.001 |
| 1985–1989 ***X*** age 70–74 | 0.779 | [0.677, 0.897] | 0.001 |
| 1985–1989 ***X*** age 75–79 | 0.789 | [0.677, 0.919] | 0.002 |
| 1985–1989 ***X*** age 80–84 | 0.859 | [0.714, 1.032] | 0.104 |
| 1985–1989 ***X*** age 85+ | 0.879 | [0.712, 1.084] | 0.229 |
| 1995–1999 ***X*** age 5–9 | 0.904 | [0.735, 1.113] | 0.342 |
| 1995–1999 ***X*** age 10–14 | 1.31 | [0.979, 1.753] | 0.069 |
| 1995–1999 ***X*** age 15–19 | 1.512 | [1.157, 1.976] | 0.002 |
| 1995–1999 ***X*** age 20–24 | 1.463 | [1.135, 1.887] | 0.003 |
| 1995–1999 ***X*** age 25–29 | 0.913 | [0.707, 1.178] | 0.484 |
| 1995–1999 ***X*** age 30–34 | 1.236 | [0.964, 1.586] | 0.095 |
| 1995–1999 ***X*** age 35–39 | 1.013 | [0.797, 1.287] | 0.917 |
| 1995–1999 ***X*** age 40–44 | 0.958 | [0.767, 1.195] | 0.702 |
| 1995–1999 ***X*** age 45–49 | 1.021 | [0.824, 1.264] | 0.852 |
| 1995–1999 ***X*** age 50–54 | 0.927 | [0.783, 1.098] | 0.382 |
| 1995–1999 ***X*** age 55–59 | 1.179 | [1.023, 1.359] | 0.023 |
| 1995–1999 ***X*** age 60–64 | 1.148 | [1.015, 1.298] | 0.028 |
| 1995–1999 ***X*** age 65–69 | 1.161 | [1.035, 1.302] | 0.011 |
| 1995–1999 ***X*** age 70–74 | 1.038 | [0.923, 1.167] | 0.53 |
| 1995–1999 ***X*** age 75–79 | 1.188 | [1.047, 1.348] | 0.008 |
| 1995–1999 ***X*** age 80–84 | 1.291 | [1.111, 1.500] | 0.001 |
| 1995–1999 ***X*** age 85+ | 1.292 | [1.096, 1.524] | 0.002 |
| 2000–2004 ***X*** age 5–9 | 1.151 | [0.928, 1.429] | 0.201 |
| 2000–2004 ***X*** age 10–14 | 1.846 | [1.381, 2.468] | < 0.001 |
| 2000–2004 ***X*** age 15–19 | 1.795 | [1.365, 2.359] | < 0.001 |
| 2000–2004 ***X*** age 20–24 | 1.517 | [1.158, 1.989] | 0.003 |
| 2000–2004 ***X*** age 25–29 | 1.052 | [0.804, 1.377] | 0.71 |
| 2000–2004 ***X*** age 30–34 | 1.301 | [0.996, 1.700] | 0.053 |
| 2000–2004 ***X*** age 35–39 | 1.23 | [0.965, 1.568] | 0.095 |
| 2000–2004 ***X*** age 40–44 | 1.191 | [0.966, 1.470] | 0.102 |
| 2000–2004 ***X*** age 45–49 | 1.339 | [1.094, 1.639] | 0.005 |
| 2000–2004 ***X*** age 50–54 | 1.089 | [0.915, 1.295] | 0.336 |
| 2000–2004 ***X*** age 55–59 | 1.197 | [1.030, 1.391] | 0.019 |
| 2000–2004 ***X*** age 60–64 | 1.387 | [1.222, 1.573] | < 0.001 |
| 2000–2004 ***X*** age 65–69 | 1.274 | [1.135, 1.431] | < 0.001 |
| 2000–2004 ***X*** age 70–74 | 1.306 | [1.163, 1.466] | < 0.001 |
| 2000–2004 ***X*** age 75–79 | 1.608 | [1.422, 1.818] | < 0.001 |
| 2000–2004 ***X*** age 80–84 | 1.746 | [1.509, 2.020] | < 0.001 |
| 2000–2004 ***X*** age 85+ | 1.654 | [1.408, 1.943] | < 0.001 |
| 2005–2009 ***X*** age 5–9 | 1.035 | [0.726, 1.476] | 0.848 |
| 2005–2009 ***X*** age 10–14 | 1.586 | [0.994, 2.529] | 0.053 |
| 2005–2009 ***X*** age 15–19 | 2.26 | [1.554, 3.286] | < 0.001 |
| 2005–2009 ***X*** age 20–24 | 1.647 | [1.103, 2.460] | 0.015 |
| 2005–2009 ***X*** age 25–29 | 1.021 | [0.663, 1.573] | 0.925 |
| 2005–2009 ***X*** age 30–34 | 1.699 | [1.162, 2.484] | 0.006 |
| 2005–2009 ***X*** age 35–39 | 1.785 | [1.291, 2.468] | < 0.001 |
| 2005–2009 ***X*** age 40–44 | 1.324 | [0.989, 1.772] | 0.059 |
| 2005–2009 ***X*** age 45–49 | 1.716 | [1.328, 2.217] | < 0.001 |
| 2005–2009 ***X*** age 50–54 | 1.566 | [1.248, 1.966] | < 0.001 |
| 2005–2009 ***X*** age 55–59 | 1.399 | [1.121, 1.745] | 0.003 |
| 2005–2009 ***X*** age 60–64 | 1.654 | [1.387, 1.972] | < 0.001 |
| 2005–2009 ***X*** age 65–69 | 1.698 | [1.444, 1.997] | < 0.001 |
| 2005–2009 ***X*** age 70–74 | 1.834 | [1.575, 2.136] | < 0.001 |
| 2005–2009 ***X*** age 75–79 | 2.075 | [1.763, 2.444] | < 0.001 |
| 2005–2009 ***X*** age 80–84 | 1.955 | [1.616, 2.366] | < 0.001 |
| 2005–2009 ***X*** age 85+ | 1.991 | [1.613, 2.458] | < 0.001 |

^a^ Logistic regression of death on sex, age, and time period. Unit of analysis is “person-year.” Explanatory variables are defined at beginning of each year.
