# Supplementary material for: The Evolving Demographic and Health Transition in Four Low- and Middle-Income Countries: Evidence from Four Sites in the INDEPTH Network of Longitudinal Health and Demographic Surveillance Systems
Source: PLoS One. 2016 Jun 15;11(6):e0157281. doi: 10.1371/journal.pone.0157281 (PMC4909223; doi:10.1371/journal.pone.0157281)
Supplement: S2 Table — (DOCX) [file pone.0157281.s007.docx]

**Table S2. Logistic regression of all-cause mortality, Agincourt, South Africa, 1994–2009 (N= 1,045,993 person years).**

| Variable | Odds Ratio | 95% CI | p-value |
| --- | --- | --- | --- |
| *Sex* |  |  |  |
| Male | 0.94 | [0.575, 1.538] | 0.806 |
| *5-Year Age Groups* |  |  |  |
| 0–4 | 1.000 | – | – |
| 5–9 | 0.081 | [0.054, 0.123] | < 0.001 |
| 10–14 | 0.101 | [0.070, 0.146] | < 0.001 |
| 15–19 | 0.172 | [0.061, 0.487] | 0.001 |
| 20–24 | 0.369 | [0.264, 0.516] | < 0.001 |
| 25–29 | 0.238 | [0.084, 0.673] | 0.007 |
| 30–34 | 1.046 | [0.858, 1.276] | 0.654 |
| 35–39 | 0.562 | [0.235, 1.343] | 0.195 |
| 40–44 | 0.907 | [0.661, 1.246] | 0.547 |
| 45–49 | 0.811 | [0.316, 2.082] | 0.663 |
| 50–54 | 0.512 | [0.157, 1.673] | 0.268 |
| 55–59 | 0.603 | [0.184, 1.970] | 0.402 |
| 60–64 | 1.281 | [0.980, 1.674] | 0.07 |
| 65–69 | 2.417 | [1.185, 4.929] | 0.015 |
| 70–74 | 5.542 | [3.096, 9.919] | < 0.001 |
| 75–79 | 2.829 | [2.150, 3.724] | < 0.001 |
| 80–84 | 18.181 | [9.462, 34.934] | < 0.001 |
| 85+ | 20.951 | [10.203, 43.021] | < 0.001 |
| *Time Period* |  |  |  |
| 1990–1994 | 0.897 | [0.616, 1.304] | 0.569 |
| 1995–1999 | 1.000 | – | – |
| 2000–2004 | 1.725 | [1.419, 2.098] | < 0.001 |
| 2005–2009 | 2.041 | [1.687, 2.470] | < 0.001 |
| *Interactions between Sex and Age* |  |  |  |
| Male ***X*** age 5–9 | 1.14 | [0.638, 2.034] | 0.659 |
| Male ***X*** age 10–14 | 1.38 | [0.645, 2.952] | 0.407 |
| Male ***X*** age 15–19 | 1.006 | [0.231, 4.381] | 0.994 |
| Male ***X*** age 20–24 | 0.625 | [0.443, 0.884] | 0.008 |
| Male ***X*** age 25–29 | 0.729 | [0.545, 0.976] | 0.034 |
| Male ***X*** age 30–34 | 1.656 | [1.066, 2.573] | 0.025 |
| Male ***X*** age 35–39 | 1.892 | [0.601, 5.954] | 0.276 |
| Male ***X*** age 40–44 | 1.286 | [0.948, 1.744] | 0.106 |
| Male ***X*** age 45–49 | 1.418 | [1.030, 1.951] | 0.032 |
| Male ***X*** age 50–54 | 5.724 | [1.481, 22.125] | 0.011 |
| Male ***X*** age 55–59 | 3.653 | [0.883, 15.107] | 0.074 |
| Male ***X*** age 60–64 | 3.535 | [1.280, 9.763] | 0.015 |
| Male ***X*** age 65–69 | 3.731 | [1.495, 9.308] | 0.005 |
| Male ***X*** age 70–74 | 1.554 | [0.672, 3.592] | 0.303 |
| Male ***X*** age 75–79 | 1.344 | [0.514, 3.516] | 0.547 |
| Male ***X*** age 80–84 | 0.95 | [0.353, 2.554] | 0.919 |
| Male ***X*** age 85+ | 1.764 | [0.597, 5.210] | 0.304 |
| *Interactions between Sex and Time* |  |  |  |
| Male ***X*** 1995–1999 | 1.148 | [0.672, 1.960] | 0.614 |
| Male ***X*** 2000–2004 | 1.238 | [0.736, 2.082] | 0.42 |
| Male ***X*** 2005–2009 | 1.136 | [0.677, 1.906] | 0.628 |
| *Interactions between Age and Time* |  |  |  |
| 1990–1994 ***X*** age 5–9 | 1.451 | [0.474, 4.442] | 0.514 |
| 1990–1994 ***X*** age 10–14 | 1.049 | [0.304, 3.622] | 0.94 |
| 1990–1994 ***X*** age 20–24 | 0.664 | [0.244, 1.804] | 0.422 |
| 1990–1994 ***X*** age 30–34 | 0.714 | [0.350, 1.456] | 0.354 |
| 1990–1994 ***X*** age 40–44 | 0.92 | [0.398, 2.125] | 0.846 |
| 1990–1994 ***X*** age 60–64 | 1.096 | [0.482, 2.490] | 0.827 |
| 1990–1994 ***X*** age 75–79 | 2.941 | [1.419, 6.097] | 0.004 |
| 1995–1999 ***X*** age 5–9 | 1.072 | [0.553, 2.075] | 0.838 |
| 1995–1999 ***X*** age 10–14 | 0.707 | [0.353, 1.413] | 0.326 |
| 1995–1999 ***X*** age 15–19 | 1.013 | [0.329, 3.115] | 0.982 |
| 1995–1999 ***X*** age 25–29 | 1.788 | [0.600, 5.334] | 0.297 |
| 1995–1999 ***X*** age 30–34 | 0.478 | [0.323, 0.708] | < 0.001 |
| 1995–1999 ***X*** age 35–39 | 1.477 | [0.587, 3.715] | 0.408 |
| 1995–1999 ***X*** age 45–49 | 0.755 | [0.269, 2.115] | 0.593 |
| 1995–1999 ***X*** age 50–54 | 1.75 | [0.501, 6.109] | 0.381 |
| 1995–1999 ***X*** age 55–59 | 1.314 | [0.373, 4.633] | 0.671 |
| 1995–1999 ***X*** age 60–64 | 1.566 | [1.037, 2.365] | 0.033 |
| 1995–1999 ***X*** age 65–69 | 0.962 | [0.447, 2.070] | 0.921 |
| 1995–1999 ***X*** age 70–74 | 0.748 | [0.391, 1.428] | 0.379 |
| 1995–1999 ***X*** age 75–79 | 1.939 | [1.312, 2.866] | 0.001 |
| 1995–1999 ***X*** age 80–84 | 0.402 | [0.188, 0.856] | 0.018 |
| 1995–1999 ***X*** age 85+ | 0.856 | [0.392, 1.870] | 0.696 |
| 2000–2004 ***X*** age 5–9 | 0.968 | [0.530, 1.769] | 0.916 |
| 2000–2004 ***X*** age 10–14 | 0.789 | [0.451, 1.380] | 0.407 |
| 2000–2004 ***X*** age 15–19 | 0.75 | [0.250, 2.250] | 0.608 |
| 2000–2004 ***X*** age 20–24 | 1.2 | [0.796, 1.809] | 0.383 |
| 2000–2004 ***X*** age 25–29 | 3.402 | [1.180, 9.805] | 0.023 |
| 2000–2004 ***X*** age 35–39 | 1.738 | [0.708, 4.263] | 0.227 |
| 2000–2004 ***X*** age 40–44 | 1.139 | [0.772, 1.680] | 0.513 |
| 2000–2004 ***X*** age 45–49 | 1.341 | [0.507, 3.548] | 0.554 |
| 2000–2004 ***X*** age 50–54 | 2.655 | [0.793, 8.890] | 0.113 |
| 2000–2004 ***X*** age 55–59 | 1.939 | [0.573, 6.557] | 0.287 |
| 2000–2004 ***X*** age 60–64 | 1.143 | [0.784, 1.668] | 0.486 |
| 2000–2004 ***X*** age 65–69 | 0.751 | [0.351, 1.604] | 0.459 |
| 2000–2004 ***X*** age 70–74 | 0.329 | [0.175, 0.620] | 0.001 |
| 2000–2004 ***X*** age 80–84 | 0.303 | [0.150, 0.610] | 0.001 |
| 2000–2004 ***X*** age 85+ | 0.35 | [0.161, 0.763] | 0.008 |
| 2005–2009 ***X*** age 15–19 | 0.633 | [0.212, 1.896] | 0.414 |
| 2005–2009 ***X*** age 20–24 | 0.833 | [0.551, 1.258] | 0.384 |
| 2005–2009 ***X*** age 25–29 | 3.413 | [1.188, 9.803] | 0.023 |
| 2005–2009 ***X*** age 30–34 | 1.076 | [0.823, 1.406] | 0.592 |
| 2005–2009 ***X*** age 35–39 | 1.866 | [0.764, 4.556] | 0.171 |
| 2005–2009 ***X*** age 40–44 | 0.996 | [0.680, 1.459] | 0.985 |
| 2005–2009 ***X*** age 45–49 | 1.376 | [0.523, 3.621] | 0.517 |
| 2005–2009 ***X*** age 50–54 | 2.768 | [0.831, 9.217] | 0.097 |
| 2005–2009 ***X*** age 55–59 | 2.137 | [0.638, 7.157] | 0.218 |
| 2005–2009 ***X*** age 65–69 | 0.631 | [0.296, 1.344] | 0.232 |
| 2005–2009 ***X*** age 70–74 | 0.402 | [0.214, 0.755] | 0.005 |
| 2005–2009 ***X*** age 75–79 | 0.842 | [0.590, 1.200] | 0.341 |
| 2005–2009 ***X*** age 80–84 | 0.208 | [0.103, 0.419] | < 0.001 |
| 2005–2009 ***X*** age 85+ | 0.341 | [0.161, 0.724] | 0.005 |
| *Interactions between Sex, Age, and Time* | |  |  |
| Male ***X*** 1990–1994 ***X*** age 5–9 | 0.462 | [0.072, 2.971] | 0.416 |
| Male ***X*** 1990–1994 ***X*** age 10–14 | 1.526 | [0.291, 7.998] | 0.617 |
| Male ***X*** 1990–1994 ***X*** age 20–24 | 1.792 | [0.451, 7.115] | 0.407 |
| Male ***X*** 1990–1994 ***X*** age 25–29 | 5.209 | [1.478, 18.353] | 0.01 |
| Male ***X*** 1990–1994 ***X*** age 30–34 | 0.465 | [0.146, 1.480] | 0.195 |
| Male ***X*** 1990–1994 ***X*** age 40–44 | 0.722 | [0.216, 2.419] | 0.598 |
| Male ***X*** 1990–1994 ***X*** age 45–49 | 2.545 | [0.793, 8.174] | 0.117 |
| Male ***X*** 1995–1999 ***X*** age 5–9 | 0.801 | [0.317, 2.026] | 0.64 |
| Male ***X*** 1995–1999 ***X*** age 15–19 | 0.641 | [0.129, 3.200] | 0.588 |
| Male ***X*** 1995–1999 ***X*** age 20–24 | 1.148 | [0.626, 2.105] | 0.655 |
| Male ***X*** 1995–1999 ***X*** age 25–29 | 1.533 | [0.885, 2.655] | 0.128 |
| Male ***X*** 1995–1999 ***X*** age 35–39 | 0.735 | [0.218, 2.482] | 0.62 |
| Male ***X*** 1995–1999 ***X*** age 40–44 | 0.977 | [0.573, 1.665] | 0.933 |
| Male ***X*** 1995–1999 ***X*** age 45–49 | 1.989 | [1.102, 3.588] | 0.022 |
| Male ***X*** 1995–1999 ***X*** age 50–54 | 0.348 | [0.082, 1.476] | 0.152 |
| Male ***X*** 1995–1999 ***X*** age 55–59 | 0.778 | [0.171, 3.531] | 0.745 |
| Male ***X*** 1995–1999 ***X*** age 60–64 | 0.349 | [0.114, 1.063] | 0.064 |
| Male ***X*** 1995–1999 ***X*** age 65–69 | 0.422 | [0.155, 1.150] | 0.091 |
| Male ***X*** 1995–1999 ***X*** age 70–74 | 0.853 | [0.336, 2.167] | 0.738 |
| Male ***X*** 1995–1999 ***X*** age 75–79 | 1.235 | [0.439, 3.477] | 0.689 |
| Male ***X*** 1995–1999 ***X*** age 80–84 | 1.263 | [0.410, 3.894] | 0.685 |
| Male ***X*** 1995–1999 ***X*** age 85+ | 0.485 | [0.148, 1.590] | 0.232 |
| Male ***X*** 2000–2004 ***X*** age 10–14 | 0.532 | [0.201, 1.411] | 0.205 |
| Male ***X*** 2000–2004 ***X*** age 15–19 | 0.808 | [0.171, 3.828] | 0.788 |
| Male ***X*** 2000–2004 ***X*** age 30–34 | 0.581 | [0.346, 0.977] | 0.041 |
| Male ***X*** 2000–2004 ***X*** age 35–39 | 0.638 | [0.196, 2.080] | 0.456 |
| Male ***X*** 2000–2004 ***X*** age 50–54 | 0.208 | [0.052, 0.834] | 0.027 |
| Male ***X*** 2000–2004 ***X*** age 55–59 | 0.507 | [0.117, 2.195] | 0.363 |
| Male ***X*** 2000–2004 ***X*** age 60–64 | 0.48 | [0.164, 1.405] | 0.18 |
| Male ***X*** 2000–2004 ***X*** age 65–69 | 0.404 | [0.150, 1.083] | 0.071 |
| Male ***X*** 2000–2004 ***X*** age 70–74 | 1.104 | [0.443, 2.751] | 0.832 |
| Male ***X*** 2000–2004 ***X*** age 75–79 | 1.537 | [0.547, 4.314] | 0.415 |
| Male ***X*** 2000–2004 ***X*** age 80–84 | 1.135 | [0.394, 3.271] | 0.815 |
| Male ***X*** 2000–2004 ***X*** age 85+ | 0.688 | [0.214, 2.205] | 0.529 |
| Male ***X*** 2005–2009 ***X*** age 5–9 | 1.134 | [0.509, 2.527] | 0.758 |
| Male ***X*** 2005–2009 ***X*** age 10–14 | 0.704 | [0.280, 1.768] | 0.455 |
| Male ***X*** 2005–2009 ***X*** age 15–19 | 0.728 | [0.153, 3.466] | 0.69 |
| Male ***X*** 2005–2009 ***X*** age 20–24 | 1.122 | [0.684, 1.841] | 0.649 |
| Male ***X*** 2005–2009 ***X*** age 25–29 | 1.01 | [0.679, 1.500] | 0.962 |
| Male ***X*** 2005–2009 ***X*** age 30–34 | 0.601 | [0.362, 0.998] | 0.049 |
| Male ***X*** 2005–2009 ***X*** age 35–39 | 0.74 | [0.228, 2.397] | 0.616 |
| Male ***X*** 2005–2009 ***X*** age 40–44 | 1.568 | [1.041, 2.363] | 0.032 |
| Male ***X*** 2005–2009 ***X*** age 45–49 | 1.032 | [0.669, 1.590] | 0.888 |
| Male ***X*** 2005–2009 ***X*** age 50–54 | 0.218 | [0.055, 0.872] | 0.031 |
| Male ***X*** 2005–2009 ***X*** age 55–59 | 0.448 | [0.105, 1.919] | 0.279 |
| Male ***X*** 2005–2009 ***X*** age 60–64 | 0.662 | [0.226, 1.938] | 0.452 |
| Male ***X*** 2005–2009 ***X*** age 65–69 | 0.604 | [0.228, 1.599] | 0.31 |
| Male ***X*** 2005–2009 ***X*** age 70–74 | 1.089 | [0.439, 2.701] | 0.855 |
| Male ***X*** 2005–2009 ***X*** age 75–79 | 1.279 | [0.460, 3.551] | 0.637 |
| Male ***X*** 2005–2009 ***X*** age 80–84 | 2.077 | [0.721, 5.983] | 0.176 |
| Male ***X*** 2005–2009 ***X*** age 85+ | 0.813 | [0.262, 2.523] | 0.72 |

^a Logistic regression of death on sex, age, and time period. Unit of analysis is “person-year.” Explanatory variables are defined at beginning of each year.^
