# Supplementary material for: The Evolving Demographic and Health Transition in Four Low- and Middle-Income Countries: Evidence from Four Sites in the INDEPTH Network of Longitudinal Health and Demographic Surveillance Systems
Source: PLoS One. 2016 Jun 15;11(6):e0157281. doi: 10.1371/journal.pone.0157281 (PMC4909223; doi:10.1371/journal.pone.0157281)
Supplement: S3 Table — (DOCX) [file pone.0157281.s008.docx]

**Table S3. Logistic regression of all-cause mortality, Navrongo, Ghana, 1995–2007 (N = 1,600,536 person years).**

| Variable | Odds Ratio | 95% CI | p-value |
| --- | --- | --- | --- |
| *Sex* |  |  |  |
| Male | 1.113 | [0.982, 1.261] | 0.095 |
| *5-Year Age Groups* |  |  |  |
| 0–4 | 1.000 | – | – |
| 5–9 | 0.12 | [0.091, 0.158] | < 0.001 |
| 10–14 | 0.069 | [0.057, 0.083] | < 0.001 |
| 15–19 | 0.073 | [0.050, 0.107] | < 0.001 |
| 20–24 | 0.118 | [0.096, 0.146] | < 0.001 |
| 25–29 | 0.161 | [0.135, 0.193] | < 0.001 |
| 30–34 | 0.284 | [0.214, 0.376] | < 0.001 |
| 35–39 | 0.315 | [0.245, 0.404] | < 0.001 |
| 40–44 | 0.19 | [0.161, 0.224] | < 0.001 |
| 45–49 | 0.27 | [0.229, 0.319] | < 0.001 |
| 50–54 | 0.385 | [0.340, 0.435] | < 0.001 |
| 55–59 | 0.67 | [0.595, 0.754] | < 0.001 |
| 60–64 | 0.795 | [0.706, 0.896] | < 0.001 |
| 65–69 | 1.769 | [1.511, 2.071] | < 0.001 |
| 70–74 | 2.019 | [1.779, 2.290] | < 0.001 |
| 75–79 | 4.318 | [3.660, 5.095] | < 0.001 |
| 80–84 | 5.769 | [4.520, 7.364] | < 0.001 |
| 85+ | 8.517 | [6.737, 10.768] | < 0.001 |
| *Time Period* |  |  |  |
| 1995–1999 | 2.275 | [2.053, 2.521] | < 0.001 |
| 2000–2004 | 1.676 | [1.506, 1.866] | < 0.001 |
| 2005–2009 | 1.000 | – | – |
| *Interactions between Sex and Age* |  |  |  |
| Male ***X*** age 5–9 | 1.394 | [1.104, 1.762] | 0.005 |
| Male ***X*** age 10–14 | 1.425 | [0.921, 2.206] | 0.112 |
| Male ***X*** age 15–19 | 1.089 | [0.666, 1.782] | 0.734 |
| Male ***X*** age 20–24 | 0.797 | [0.589, 1.079] | 0.142 |
| Male ***X*** age 25–29 | 0.955 | [0.726, 1.256] | 0.742 |
| Male ***X*** age 30–34 | 1.618 | [1.125, 2.328] | 0.009 |
| Male ***X*** age 35–39 | 1.864 | [1.486, 2.340] | < 0.001 |
| Male ***X*** age 40–44 | 2.199 | [1.773, 2.727] | < 0.001 |
| Male ***X*** age 45–49 | 1.897 | [1.432, 2.514] | < 0.001 |
| Male ***X*** age 50–54 | 1.485 | [1.246, 1.770] | < 0.001 |
| Male ***X*** age 55–59 | 1.767 | [1.373, 2.274] | < 0.001 |
| Male ***X*** age 60–64 | 1.626 | [1.285, 2.059] | < 0.001 |
| Male ***X*** age 65–69 | 1.502 | [1.210, 1.863] | < 0.001 |
| Male ***X*** age 70–74 | 0.709 | [0.589, 0.853] | < 0.001 |
| Male ***X*** age 75–79 | 1.024 | [0.830, 1.264] | 0.824 |
| Male ***X*** age 80–84 | 0.742 | [0.554, 0.994] | 0.045 |
| Male ***X*** age 85+ | 0.803 | [0.609, 1.058] | 0.119 |
| *Interactions between Sex and Time* |  |  |  |
| Male ***X*** 1995–1999 | 0.938 | [0.815, 1.080] | 0.373 |
| Male ***X*** 2000–2004 | 0.897 | [0.774, 1.039] | 0.147 |
| *Interactions between Age and Time* |  |  |  |
| 1995–1999 ***X*** age 5–9 | 0.848 | [0.621, 1.158] | 0.3 |
| 1995–1999 ***X*** age 15–19 | 0.962 | [0.617, 1.501] | 0.866 |
| 1995–1999 ***X*** age 30–34 | 0.614 | [0.443, 0.852] | 0.003 |
| 1995–1999 ***X*** age 35–39 | 0.447 | [0.330, 0.606] | < 0.001 |
| 1995–1999 ***X*** age 45–49 | 0.899 | [0.721, 1.122] | 0.348 |
| 1995–1999 ***X*** age 55–59 | 0.899 | [0.768, 1.053] | 0.188 |
| 1995–1999 ***X*** age 65–69 | 0.745 | [0.616, 0.899] | 0.002 |
| 1995–1999 ***X*** age 75–79 | 0.62 | [0.503, 0.764] | < 0.001 |
| 1995–1999 ***X*** age 80–84 | 0.555 | [0.401, 0.767] | < 0.001 |
| 1995–1999 ***X*** age 85+ | 0.484 | [0.353, 0.664] | < 0.001 |
| 2000–2004 ***X*** age 5–9 | 0.817 | [0.589, 1.133] | 0.226 |
| 2000–2004 ***X*** age 10–14 | 1.071 | [0.812, 1.414] | 0.627 |
| 2000–2004 ***X*** age 15–19 | 1.291 | [0.832, 2.002] | 0.254 |
| 2000–2004 ***X*** age 20–24 | 1.412 | [1.056, 1.888] | 0.02 |
| 2000–2004 ***X*** age 25–29 | 1.202 | [0.920, 1.572] | 0.177 |
| 2000–2004 ***X*** age 30–34 | 0.814 | [0.585, 1.134] | 0.225 |
| 2000–2004 ***X*** age 35–39 | 0.762 | [0.563, 1.031] | 0.078 |
| 2000–2004 ***X*** age 40–44 | 1.394 | [1.114, 1.744] | 0.004 |
| 2000–2004 ***X*** age 50–54 | 1.04 | [0.863, 1.254] | 0.682 |
| 2000–2004 ***X*** age 60–64 | 1.345 | [1.149, 1.575] | < 0.001 |
| 2000–2004 ***X*** age 65–69 | 0.883 | [0.726, 1.074] | 0.214 |
| 2000–2004 ***X*** age 70–74 | 0.892 | [0.749, 1.061] | 0.196 |
| 2000–2004 ***X*** age 75–79 | 0.672 | [0.536, 0.842] | 0.001 |
| 2000–2004 ***X*** age 80–84 | 0.686 | [0.511, 0.921] | 0.012 |
| 2000–2004 ***X*** age 85+ | 0.713 | [0.519, 0.978] | 0.036 |
| 2005–2009 ***X*** age 10–14 | 1.051 | [0.709, 1.560] | 0.804 |
| 2005–2009 ***X*** age 20–24 | 1.264 | [0.856, 1.866] | 0.239 |
| 2005–2009 ***X*** age 25–29 | 1.486 | [1.057, 2.090] | 0.023 |
| 2005–2009 ***X*** age 40–44 | 1.106 | [0.785, 1.558] | 0.565 |
| 2005–2009 ***X*** age 45–49 | 1.511 | [1.156, 1.975] | 0.003 |
| 2005–2009 ***X*** age 50–54 | 1.422 | [1.112, 1.820] | 0.005 |
| 2005–2009 ***X*** age 55–59 | 1.128 | [0.904, 1.408] | 0.286 |
| 2005–2009 ***X*** age 60–64 | 1.431 | [1.164, 1.759] | 0.001 |
| 2005–2009 ***X*** age 70–74 | 1.313 | [1.055, 1.634] | 0.015 |
| *Interactions between Sex, Age, and Time* | |  |  |
| Male ***X*** 1995–1999 ***X*** age 5–9 | 0.763 | [0.560, 1.038] | 0.085 |
| Male ***X*** 1995–1999 ***X*** age 10–14 | 0.757 | [0.459, 1.248] | 0.275 |
| Male ***X*** 1995–1999 ***X*** age 15–19 | 0.996 | [0.562, 1.766] | 0.989 |
| Male ***X*** 1995–1999 ***X*** age 30–34 | 0.817 | [0.529, 1.262] | 0.362 |
| Male ***X*** 1995–1999 ***X*** age 35–39 | 1.016 | [0.735, 1.406] | 0.922 |
| Male ***X*** 1995–1999 ***X*** age 45–49 | 0.984 | [0.697, 1.388] | 0.926 |
| Male ***X*** 1995–1999 ***X*** age 55–59 | 0.678 | [0.505, 0.911] | 0.01 |
| Male ***X*** 1995–1999 ***X*** age 60–64 | 0.782 | [0.586, 1.044] | 0.095 |
| Male ***X*** 1995–1999 ***X*** age 65–69 | 0.651 | [0.500, 0.847] | 0.001 |
| Male ***X*** 1995–1999 ***X*** age 75–79 | 0.872 | [0.660, 1.151] | 0.332 |
| Male ***X*** 1995–1999 ***X*** age 85+ | 0.995 | [0.675, 1.466] | 0.979 |
| Male ***X*** 2000–2004 ***X*** age 10–14 | 0.852 | [0.509, 1.425] | 0.542 |
| Male ***X*** 2000–2004 ***X*** age 15–19 | 1.05 | [0.596, 1.851] | 0.866 |
| Male ***X*** 2000–2004 ***X*** age 20–24 | 1.067 | [0.706, 1.614] | 0.757 |
| Male ***X*** 2000–2004 ***X*** age 25–29 | 1.017 | [0.683, 1.513] | 0.936 |
| Male ***X*** 2000–2004 ***X*** age 30–34 | 0.887 | [0.571, 1.379] | 0.595 |
| Male ***X*** 2000–2004 ***X*** age 40–44 | 0.849 | [0.630, 1.145] | 0.284 |
| Male ***X*** 2000–2004 ***X*** age 45–49 | 1.07 | [0.748, 1.532] | 0.71 |
| Male ***X*** 2000–2004 ***X*** age 50–54 | 1.397 | [1.080, 1.807] | 0.011 |
| Male ***X*** 2000–2004 ***X*** age 55–59 | 0.909 | [0.671, 1.232] | 0.538 |
| Male ***X*** 2000–2004 ***X*** age 60–64 | 0.832 | [0.629, 1.101] | 0.198 |
| Male ***X*** 2000–2004 ***X*** age 65–69 | 0.682 | [0.518, 0.898] | 0.006 |
| Male ***X*** 2000–2004 ***X*** age 70–74 | 1.652 | [1.285, 2.124] | < 0.001 |
| Male ***X*** 2000–2004 ***X*** age 80–84 | 1.156 | [0.796, 1.679] | 0.445 |
| Male ***X*** 2005–2009 ***X*** age 5–9 | 0.687 | [0.442, 1.067] | 0.095 |
| Male ***X*** 2005–2009 ***X*** age 20–24 | 1.029 | [0.596, 1.776] | 0.918 |
| Male ***X*** 2005–2009 ***X*** age 25–29 | 0.93 | [0.569, 1.522] | 0.773 |
| Male ***X*** 2005–2009 ***X*** age 35–39 | 0.946 | [0.632, 1.417] | 0.788 |
| Male ***X*** 2005–2009 ***X*** age 40–44 | 1.41 | [0.924, 2.152] | 0.111 |
| Male ***X*** 2005–2009 ***X*** age 50–54 | 1.391 | [0.999, 1.937] | 0.051 |
| Male ***X*** 2005–2009 ***X*** age 70–74 | 1.849 | [1.361, 2.511] | < 0.001 |
| Male ***X*** 2005–2009 ***X*** age 75–79 | 0.873 | [0.635, 1.200] | 0.402 |
| Male ***X*** 2005–2009 ***X*** age 80–84 | 1.31 | [0.844, 2.035] | 0.228 |
| Male ***X*** 2005–2009 ***X*** age 85+ | 1.082 | [0.713, 1.640] | 0.712 |

^a Logistic regression of death on sex, age, and time period. Unit of analysis is “person-year.” Explanatory variables are defined at beginning of each year.^
