# Supplementary material for: The Evolving Demographic and Health Transition in Four Low- and Middle-Income Countries: Evidence from Four Sites in the INDEPTH Network of Longitudinal Health and Demographic Surveillance Systems
Source: PLoS One. 2016 Jun 15;11(6):e0157281. doi: 10.1371/journal.pone.0157281 (PMC4909223; doi:10.1371/journal.pone.0157281)
Supplement: S4 Table — (DOCX) [file pone.0157281.s009.docx]

**Table S4. Logistic regression of all-cause mortality, Filabavi, Vietnam, 1999–2007 (N = 425,225 person years).**

| Variable | Odds Ratio | 95% CI | p-value |
| --- | --- | --- | --- |
| *Sex* |  |  |  |
| Male | 1.655 | [1.175, 2.332] | 0.004 |
| *5-Year Age Groups* |  |  |  |
| 0–4 | 1.000 | – | – |
| 5–9 | 0.145 | [0.069, 0.305] | < 0.001 |
| 10–14 | 0.142 | [0.072, 0.280] | < 0.001 |
| 15–19 | 0.24 | [0.139, 0.416] | < 0.001 |
| 20–24 | 0.403 | [0.242, 0.670] | < 0.001 |
| 25–29 | 0.277 | [0.151, 0.510] | < 0.001 |
| 30–34 | 0.26 | [0.139, 0.489] | < 0.001 |
| 35–39 | 0.225 | [0.117, 0.433] | < 0.001 |
| 40–44 | 0.449 | [0.274, 0.735] | 0.001 |
| 45–49 | 0.648 | [0.406, 1.034] | 0.069 |
| 50–54 | 1.021 | [0.647, 1.613] | 0.927 |
| 55–59 | 1.126 | [0.688, 1.844] | 0.636 |
| 60–64 | 1.947 | [1.278, 2.966] | 0.002 |
| 65–69 | 2.898 | [1.993, 4.214] | < 0.001 |
| 70–74 | 5.313 | [3.797, 7.433] | < 0.001 |
| 75–79 | 10.842 | [7.913, 14.856] | < 0.001 |
| 80–84 | 14.391 | [10.457, 19.805] | < 0.001 |
| 85+ | 44.579 | [33.104, 60.032] | < 0.001 |
| *Time Period* |  |  |  |
| 1995–1999 | 1.211 | [1.049, 1.397] | 0.009 |
| 2000–2004 | 1.06 | [0.971, 1.158] | 0.193 |
| 2005–2009 | 1.000 | – | – |
| *Interactions between Sex and Age* |  |  |  |
| Male ***X*** age 5–9 | 1.187 | [0.479, 2.943] | 0.711 |
| Male ***X*** age 10–14 | 0.859 | [0.360, 2.051] | 0.732 |
| Male ***X*** age 15–19 | 0.699 | [0.338, 1.445] | 0.334 |
| Male ***X*** age 20–24 | 0.72 | [0.365, 1.421] | 0.344 |
| Male ***X*** age 25–29 | 1.689 | [0.818, 3.490] | 0.157 |
| Male ***X*** age 30–34 | 1.685 | [0.796, 3.567] | 0.172 |
| Male ***X*** age 35–39 | 3.063 | [1.468, 6.392] | 0.003 |
| Male ***X*** age 40–44 | 1.79 | [0.994, 3.223] | 0.052 |
| Male ***X*** age 45–49 | 1.878 | [1.075, 3.282] | 0.027 |
| Male ***X*** age 50–54 | 1.396 | [0.795, 2.452] | 0.246 |
| Male ***X*** age 55–59 | 1.957 | [1.088, 3.520] | 0.025 |
| Male ***X*** age 60–64 | 1.288 | [0.759, 2.184] | 0.348 |
| Male ***X*** age 65–69 | 1.961 | [1.238, 3.107] | 0.004 |
| Male ***X*** age 70–74 | 1.586 | [1.038, 2.422] | 0.033 |
| Male ***X*** age 75–79 | 1.232 | [0.820, 1.851] | 0.315 |
| Male ***X*** age 80–84 | 1.355 | [0.889, 2.065] | 0.158 |
| Male ***X*** age 85+ | 0.935 | [0.621, 1.407] | 0.747 |

^a Logistic regression of death on sex, age, and time period. Unit of analysis is “person-year.” Explanatory variables are defined at beginning of each year.^
