# Supplementary material for: The Evolving Demographic and Health Transition in Four Low- and Middle-Income Countries: Evidence from Four Sites in the INDEPTH Network of Longitudinal Health and Demographic Surveillance Systems
Source: PLoS One. 2016 Jun 15;11(6):e0157281. doi: 10.1371/journal.pone.0157281 (PMC4909223; doi:10.1371/journal.pone.0157281)
Supplement: S5 Table — (DOCX) [file pone.0157281.s010.docx]

**Table S5. Multinomial logistic regression of cause-specific mortality, Matlab, Bangladesh, 1987–2006 (N = 4,111,333 person years).**

| Variable | Odds Ratio | 95% CI | p-value |
| --- | --- | --- | --- |
| **Communicable** |  |  |  |
| *Sex* |  |  |  |
| Male | 0.866 | [0.797, 0.942] | 0.001 |
| *10-Year Age Groups* |  |  |  |
| 0–4 | 1.000 | – | – |
| 5–9 | 0.045 | [0.035, 0.059] | < 0.001 |
| 10–19 | 0.012 | [0.008, 0.017] | < 0.001 |
| 20–29 | 0.04 | [0.031, 0.052] | < 0.001 |
| 30–39 | 0.027 | [0.018, 0.040] | < 0.001 |
| 40–49 | 0.032 | [0.022, 0.045] | < 0.001 |
| 50–59 | 0.068 | [0.053, 0.088] | < 0.001 |
| 60–69 | 0.144 | [0.113, 0.182] | < 0.001 |
| 70–79 | 0.33 | [0.255, 0.427] | < 0.001 |
| 80+ | 0.782 | [0.552, 1.107] | 0.166 |
| *Time Period* |  |  |  |
| 1985–1989 | 1.000 | – | – |
| 1990–1994 | 0.777 | [0.717, 0.841] | < 0.001 |
| 1995–1999 | 0.554 | [0.507, 0.605] | < 0.001 |
| 2000–2004 | 0.396 | [0.360, 0.436] | < 0.001 |
| 2005–2009 | 0.31 | [0.268, 0.358] | < 0.001 |
| *Interactions between Sex and Age* |  |  |  |
| Male ***X*** age 5–9 | 0.817 | [0.639, 1.045] | 0.107 |
| Male ***X*** age 10–19 | 0.744 | [0.550, 1.005] | 0.054 |
| Male ***X*** age 20–29 | 0.338 | [0.251, 0.454] | < 0.001 |
| Male ***X*** age 30–39 | 0.549 | [0.414, 0.730] | < 0.001 |
| Male ***X*** age 40–49 | 1.316 | [0.997, 1.739] | 0.053 |
| Male ***X*** age 50–59 | 1.931 | [1.574, 2.369] | < 0.001 |
| Male ***X*** age 60–69 | 1.578 | [1.343, 1.854] | < 0.001 |
| Male ***X*** age 70–79 | 1.148 | [0.987, 1.336] | 0.073 |
| Male ***X*** age 80+ | 0.89 | [0.731, 1.083] | 0.245 |
| *Interactions between Sex and Time* |  |  |  |
| Male ***X*** 1990–1994 | 1.198 | [1.078, 1.333] | 0.001 |
| Male ***X*** 1995–1999 | 1.153 | [1.027, 1.294] | 0.016 |
| Male ***X*** 2000–2004 | 1.254 | [1.109, 1.417] | < 0.001 |
| Male ***X*** 2005–2009 | 1.272 | [1.063, 1.521] | 0.009 |
| *Interactions between Age and Time* |  |  |  |
| 1990–1994 ***X*** age 5–9 | 1.006 | [0.739, 1.370] | 0.97 |
| 1990–1994 ***X*** age 10–19 | 1.143 | [0.716, 1.823] | 0.575 |
| 1990–1994 ***X*** age 20–29 | 0.99 | [0.703, 1.392] | 0.952 |
| 1990–1994 ***X*** age 30–39 | 1.722 | [1.107, 2.677] | 0.016 |
| 1990–1994 ***X*** age 40–49 | 1.103 | [0.723, 1.682] | 0.649 |
| 1990–1994 ***X*** age 50–59 | 1.078 | [0.811, 1.433] | 0.605 |
| 1990–1994 ***X*** age 60–69 | 1.377 | [1.060, 1.788] | 0.017 |
| 1990–1994 ***X*** age 70–79 | 1.732 | [1.302, 2.303] | < 0.001 |
| 1990–1994 ***X*** age 80+ | 1.631 | [1.108, 2.402] | 0.013 |
| 1995–1999 ***X*** age 5–9 | 0.724 | [0.502, 1.045] | 0.085 |
| 1995–1999 ***X*** age 10–19 | 1.8 | [1.144, 2.834] | 0.011 |
| 1995–1999 ***X*** age 20–29 | 1.178 | [0.823, 1.687] | 0.37 |
| 1995–1999 ***X*** age 30–39 | 1.573 | [0.993, 2.491] | 0.054 |
| 1995–1999 ***X*** age 40–49 | 1.263 | [0.821, 1.944] | 0.288 |
| 1995–1999 ***X*** age 50–59 | 1.246 | [0.926, 1.676] | 0.146 |
| 1995–1999 ***X*** age 60–69 | 1.724 | [1.327, 2.240] | < 0.001 |
| 1995–1999 ***X*** age 70–79 | 2.246 | [1.691, 2.984] | < 0.001 |
| 1995–1999 ***X*** age 80+ | 2.144 | [1.457, 3.153] | < 0.001 |
| 2000–2004 ***X*** age 5–9 | 0.546 | [0.346, 0.862] | 0.009 |
| 2000–2004 ***X*** age 10–19 | 1.491 | [0.904, 2.461] | 0.118 |
| 2000–2004 ***X*** age 20–29 | 0.947 | [0.626, 1.434] | 0.798 |
| 2000–2004 ***X*** age 30–39 | 1.465 | [0.898, 2.391] | 0.126 |
| 2000–2004 ***X*** age 40–49 | 1.147 | [0.738, 1.784] | 0.542 |
| 2000–2004 ***X*** age 50–59 | 1.032 | [0.744, 1.432] | 0.849 |
| 2000–2004 ***X*** age 60–69 | 1.632 | [1.242, 2.142] | < 0.001 |
| 2000–2004 ***X*** age 70–79 | 2.523 | [1.898, 3.353] | < 0.001 |
| 2000–2004 ***X*** age 80+ | 3.067 | [2.105, 4.468] | < 0.001 |
| 2005–2009 ***X*** age 5–9 | 0.857 | [0.469, 1.565] | 0.615 |
| 2005–2009 ***X*** age 10–19 | 1.49 | [0.720, 3.084] | 0.283 |
| 2005–2009 ***X*** age 20–29 | 1.016 | [0.550, 1.874] | 0.961 |
| 2005–2009 ***X*** age 30–39 | 1.742 | [0.916, 3.311] | 0.09 |
| 2005–2009 ***X*** age 40–49 | 1.022 | [0.554, 1.888] | 0.944 |
| 2005–2009 ***X*** age 50–59 | 0.937 | [0.576, 1.523] | 0.792 |
| 2005–2009 ***X*** age 60–69 | 1.2 | [0.808, 1.781] | 0.367 |
| 2005–2009 ***X*** age 70–79 | 2.567 | [1.825, 3.610] | < 0.001 |
| 2005–2009 ***X*** age 80+ | 2.74 | [1.751, 4.288] | < 0.001 |
| **Noncommunicable** |  |  |  |
| *Sex* |  |  |  |
| Male | 1.086 | [0.863, 1.365] | 0.481 |
| *10-Year Age Groups* |  |  |  |
| 0–4 | 1.000 | – | – |
| 5–9 | 0.145 | [0.073, 0.291] | < 0.001 |
| 10–19 | 0.228 | [0.139, 0.376] | < 0.001 |
| 20–29 | 0.576 | [0.382, 0.868] | 0.008 |
| 30–39 | 0.794 | [0.526, 1.198] | 0.271 |
| 40–49 | 1.682 | [1.192, 2.373] | 0.003 |
| 50–59 | 4.57 | [3.390, 6.162] | < 0.001 |
| 60–69 | 9.398 | [7.011, 12.597] | < 0.001 |
| 70–79 | 14.097 | [10.242, 19.402] | < 0.001 |
| 80+ | 19.764 | [13.130, 29.750] | < 0.001 |
| *Time Period* |  |  |  |
| 1985–1989 | 1.000 | – | – |
| 1990–1994 | 1.167 | [0.857, 1.589] | 0.328 |
| 1995–1999 | 0.911 | [0.657, 1.263] | 0.577 |
| 2000–2004 | 1.074 | [0.783, 1.474] | 0.656 |
| 2005–2009 | 0.981 | [0.652, 1.476] | 0.927 |
| *Interactions between Sex and Age* |  |  |  |
| Male X age 5–9 | 1.375 | [0.877, 2.157] | 0.165 |
| Male X age 10–19 | 0.971 | [0.694, 1.360] | 0.866 |
| Male X age 20–29 | 0.954 | [0.693, 1.312] | 0.772 |
| Male X age 30–39 | 1.299 | [0.987, 1.708] | 0.062 |
| Male X age 40–49 | 1.792 | [1.412, 2.275] | < 0.001 |
| Male X age 50–59 | 1.976 | [1.587, 2.459] | < 0.001 |
| Male X age 60–69 | 1.72 | [1.397, 2.119] | < 0.001 |
| Male X age 70–79 | 1.296 | [1.050, 1.600] | 0.016 |
| Male X age 80+ | 1.202 | [0.958, 1.507] | 0.112 |
| *Interactions between Sex and Time* |  |  |  |
| Male X 1990–1994 | 0.842 | [0.712, 0.994] | 0.043 |
| Male X 1995–1999 | 0.965 | [0.819, 1.136] | 0.666 |
| Male X 2000–2004 | 0.857 | [0.733, 1.002] | 0.053 |
| Male X 2005–2009 | 0.921 | [0.777, 1.090] | 0.338 |
| *Interactions between Age and Time* |  |  |  |
| 1990–1994 X age 5–9 | 0.837 | [0.371, 1.891] | 0.669 |
| 1990–1994 X age 10–19 | 0.88 | [0.487, 1.589] | 0.671 |
| 1990–1994 X age 20–29 | 0.799 | [0.491, 1.301] | 0.367 |
| 1990–1994 X age 30–39 | 0.828 | [0.513, 1.339] | 0.442 |
| 1990–1994 X age 40–49 | 1.15 | [0.775, 1.709] | 0.487 |
| 1990–1994 X age 50–59 | 1.056 | [0.750, 1.486] | 0.756 |
| 1990–1994 X age 60–69 | 1.252 | [0.895, 1.751] | 0.19 |
| 1990–1994 X age 70–79 | 1.605 | [1.116, 2.309] | 0.011 |
| 1990–1994 X age 80+ | 1.563 | [0.986, 2.479] | 0.058 |
| 1995–1999 X age 5–9 | 1.396 | [0.637, 3.060] | 0.405 |
| 1995–1999 X age 10–19 | 1.026 | [0.565, 1.862] | 0.933 |
| 1995–1999 X age 20–29 | 0.825 | [0.494, 1.376] | 0.46 |
| 1995–1999 X age 30–39 | 1.256 | [0.781, 2.019] | 0.347 |
| 1995–1999 X age 40–49 | 1.172 | [0.778, 1.765] | 0.447 |
| 1995–1999 X age 50–59 | 1.079 | [0.754, 1.545] | 0.678 |
| 1995–1999 X age 60–69 | 1.561 | [1.100, 2.214] | 0.013 |
| 1995–1999 X age 70–79 | 2.191 | [1.507, 3.187] | < 0.001 |
| 1995–1999 X age 80+ | 2.9 | [1.832, 4.591] | < 0.001 |
| 2000–2004 X age 5–9 | 1.938 | [0.915, 4.105] | 0.084 |
| 2000–2004 X age 10–19 | 1.785 | [1.028, 3.100] | 0.039 |
| 2000–2004 X age 20–29 | 0.896 | [0.547, 1.469] | 0.663 |
| 2000–2004 X age 30–39 | 1.257 | [0.790, 2.002] | 0.335 |
| 2000–2004 X age 40–49 | 1.334 | [0.902, 1.972] | 0.149 |
| 2000–2004 X age 50–59 | 1.134 | [0.800, 1.607] | 0.479 |
| 2000–2004 X age 60–69 | 1.809 | [1.289, 2.539] | 0.001 |
| 2000–2004 X age 70–79 | 3.243 | [2.258, 4.658] | < 0.001 |
| 2000–2004 X age 80+ | 5.024 | [3.225, 7.828] | < 0.001 |
| 2005–2009 X age 5–9 | 2.041 | [0.832, 5.003] | 0.119 |
| 2005–2009 X age 10–19 | 1.729 | [0.873, 3.425] | 0.116 |
| 2005–2009 X age 20–29 | 0.976 | [0.518, 1.837] | 0.94 |
| 2005–2009 X age 30–39 | 1.751 | [1.001, 3.063] | 0.05 |
| 2005–2009 X age 40–49 | 1.484 | [0.916, 2.404] | 0.109 |
| 2005–2009 X age 50–59 | 1.283 | [0.823, 2.001] | 0.271 |
| 2005–2009 X age 60–69 | 2.196 | [1.429, 3.374] | < 0.001 |
| 2005–2009 X age 70–79 | 4.442 | [2.844, 6.938] | < 0.001 |
| 2005–2009 X age 80+ | 6.593 | [3.933, 11.052] | < 0.001 |
| **Injuries** |  |  |  |
| *Sex* |  |  |  |
| Male | 1.104 | [0.889, 1.371] | 0.372 |
| *10-Year Age Groups* |  |  |  |
| 0–4 | 1.000 | – | – |
| 5–9 | 0.149 | [0.099, 0.225] | < 0.001 |
| 10–19 | 0.088 | [0.058, 0.133] | < 0.001 |
| 20–29 | 0.135 | [0.093, 0.197] | < 0.001 |
| 30–39 | 0.056 | [0.030, 0.104] | < 0.001 |
| 40–49 | 0.065 | [0.035, 0.124] | < 0.001 |
| 50–59 | 0.108 | [0.060, 0.193] | < 0.001 |
| 60–69 | 0.192 | [0.101, 0.366] | < 0.001 |
| 70–79 | 0.446 | [0.215, 0.926] | 0.03 |
| 80+ | 0.164 | [0.022, 1.201] | 0.075 |
| *Time Period* |  |  |  |
| 1985–1989 | 1.000 | – | – |
| 1990–1994 | 0.67 | [0.529, 0.848] | 0.001 |
| 1995–1999 | 0.891 | [0.709, 1.119] | 0.32 |
| 2000–2004 | 0.853 | [0.679, 1.071] | 0.17 |
| 2005–2009 | 0.803 | [0.594, 1.085] | 0.153 |
| *Interactions between Sex and Age* |  |  |  |
| Male ***X*** age 5–9 | 1.405 | [1.012, 1.949] | 0.042 |
| Male ***X*** age 10–19 | 1.022 | [0.767, 1.363] | 0.881 |
| Male ***X*** age 20–29 | 1.403 | [1.068, 1.845] | 0.015 |
| Male ***X*** age 30–39 | 2.279 | [1.562, 3.326] | < 0.001 |
| Male ***X*** age 40–49 | 2.679 | [1.731, 4.147] | < 0.001 |
| Male ***X*** age 50–59 | 2.938 | [1.820, 4.744] | < 0.001 |
| Male ***X*** age 60–69 | 1.148 | [0.787, 1.676] | 0.474 |
| Male ***X*** age 70–79 | 0.708 | [0.481, 1.041] | 0.079 |
| Male ***X*** age 80+ | 1.125 | [0.691, 1.832] | 0.636 |
| *Interactions between Sex and Time* |  |  |  |
| Male ***X*** 1990–1994 | 1.085 | [0.828, 1.423] | 0.553 |
| Male ***X*** 1995–1999 | 1.121 | [0.862, 1.458] | 0.393 |
| Male ***X*** 2000–2004 | 1.074 | [0.828, 1.394] | 0.59 |
| Male ***X*** 2005–2009 | 0.755 | [0.539, 1.058] | 0.102 |
| *Interactions between Age and Time* |  |  |  |
| 1990–1994 ***X*** age 5–9 | 0.926 | [0.567, 1.513] | 0.759 |
| 1990–1994 ***X*** age 10–19 | 1.309 | [0.792, 2.163] | 0.294 |
| 1990–1994 ***X*** age 20–29 | 1.686 | [1.097, 2.591] | 0.017 |
| 1990–1994 ***X*** age 30–39 | 1.735 | [0.888, 3.391] | 0.107 |
| 1990–1994 ***X*** age 40–49 | 1.708 | [0.858, 3.402] | 0.128 |
| 1990–1994 ***X*** age 50–59 | 1.004 | [0.540, 1.866] | 0.99 |
| 1990–1994 ***X*** age 60–69 | 1.762 | [0.850, 3.654] | 0.128 |
| 1990–1994 ***X*** age 70–79 | 1.827 | [0.788, 4.238] | 0.16 |
| 1990–1994 ***X*** age 80+ | 3.453 | [0.400, 29.815] | 0.26 |
| 1995–1999 ***X*** age 5–9 | 0.623 | [0.377, 1.029] | 0.064 |
| 1995–1999 ***X*** age 10–19 | 1.378 | [0.862, 2.203] | 0.18 |
| 1995–1999 ***X*** age 20–29 | 1.265 | [0.825, 1.941] | 0.281 |
| 1995–1999 ***X*** age 30–39 | 1.486 | [0.778, 2.838] | 0.23 |
| 1995–1999 ***X*** age 40–49 | 0.913 | [0.451, 1.848] | 0.8 |
| 1995–1999 ***X*** age 50–59 | 0.639 | [0.339, 1.204] | 0.165 |
| 1995–1999 ***X*** age 60–69 | 1.301 | [0.639, 2.648] | 0.468 |
| 1995–1999 ***X*** age 70–79 | 1.347 | [0.593, 3.059] | 0.477 |
| 1995–1999 ***X*** age 80+ | 6.236 | [0.813, 47.832] | 0.078 |
| 2000–2004 ***X*** age 5–9 | 1.195 | [0.756, 1.890] | 0.446 |
| 2000–2004 ***X*** age 10–19 | 1.379 | [0.857, 2.217] | 0.185 |
| 2000–2004 ***X*** age 20–29 | 1.163 | [0.749, 1.807] | 0.501 |
| 2000–2004 ***X*** age 30–39 | 1.471 | [0.764, 2.829] | 0.248 |
| 2000–2004 ***X*** age 40–49 | 1.121 | [0.578, 2.177] | 0.735 |
| 2000–2004 ***X*** age 50–59 | 0.504 | [0.257, 0.988] | 0.046 |
| 2000–2004 ***X*** age 60–69 | 1.524 | [0.761, 3.054] | 0.234 |
| 2000–2004 ***X*** age 70–79 | 2.142 | [0.987, 4.649] | 0.054 |
| 2000–2004 ***X*** age 80+ | 13.53 | [1.836, 99.716] | 0.011 |
| 2005–2009 ***X*** age 5–9 | 0.744 | [0.361, 1.535] | 0.424 |
| 2005–2009 ***X*** age 10–19 | 1.782 | [0.980, 3.240] | 0.058 |
| 2005–2009 ***X*** age 20–29 | 0.981 | [0.520, 1.852] | 0.953 |
| 2005–2009 ***X*** age 30–39 | 1.719 | [0.763, 3.872] | 0.191 |
| 2005–2009 ***X*** age 40–49 | 1.364 | [0.613, 3.033] | 0.446 |
| 2005–2009 ***X*** age 50–59 | 1.042 | [0.476, 2.278] | 0.919 |
| 2005–2009 ***X*** age 60–69 | 2.044 | [0.917, 4.555] | 0.08 |
| 2005–2009 ***X*** age 70–79 | 2.714 | [1.160, 6.348] | 0.021 |
| 2005–2009 ***X*** age 80+ | 22.06 | [2.915, 166.920] | 0.003 |

^a Multinomial logistic regression of adult death by cause on sex, age, and time period. Unit of analysis is “person-year.” Explanatory variables are defined at beginning of each year. Referent group is surviving adults.^
