# Supplementary material for: The Evolving Demographic and Health Transition in Four Low- and Middle-Income Countries: Evidence from Four Sites in the INDEPTH Network of Longitudinal Health and Demographic Surveillance Systems
Source: PLoS One. 2016 Jun 15;11(6):e0157281. doi: 10.1371/journal.pone.0157281 (PMC4909223; doi:10.1371/journal.pone.0157281)
Supplement: S6 Table — (DOCX) [file pone.0157281.s011.docx]

**Table S6. Multinomial logistic regression of cause-specific mortality, Agincourt, South Africa, 1994–2004 (N = 712,392 person years).**

| Variable | Odds Ratio | 95% CI | p-value |
| --- | --- | --- | --- |
| **HIV/TB** |  |  |  |
| *Sex* |  |  |  |
| Male | 0.872 | [0.671, 1.132] | 0.304 |
| *10-Year Age Groups* |  |  |  |
| 0–4 | 1.000 | – | – |
| 5–9 | 0.023 | [0.007, 0.071] | < 0.001 |
| 10–19 | 0.071 | [0.044, 0.117] | < 0.001 |
| 20–29 | 0.89 | [0.703, 1.128] | 0.336 |
| 30–39 | 1.51 | [1.203, 1.895] | < 0.001 |
| 40–49 | 1.307 | [1.009, 1.692] | 0.042 |
| 50–59 | 0.632 | [0.430, 0.929] | 0.02 |
| 60–69 | 0.534 | [0.339, 0.842] | 0.007 |
| 70–79 | 0.272 | [0.133, 0.557] | < 0.001 |
| 80+ | 0.752 | [0.350, 1.612] | 0.463 |
| *Time Period* |  |  |  |
| 1990–1994 | 1.000 | – | – |
| 1995–1999 | 2.597 | [1.743, 3.868] | < 0.001 |
| 2000–2004 | 7.478 | [5.067, 11.037] | < 0.001 |
| *Interactions between Sex and Age* | |  |  |
| Male ***X*** age 5–9 | 2.294 | [0.560, 9.398] | 0.249 |
| Male ***X*** age 10–19 | 0.613 | [0.271, 1.387] | 0.24 |
| Male ***X*** age 20–29 | 0.648 | [0.450, 0.935] | 0.02 |
| Male ***X*** age 30–39 | 1.403 | [1.012, 1.946] | 0.042 |
| Male ***X*** age 40–49 | 2.114 | [1.482, 3.017] | < 0.001 |
| Male ***X*** age 50–59 | 3.521 | [2.178, 5.695] | < 0.001 |
| Male ***X*** age 60–69 | 4.408 | [2.512, 7.735] | < 0.001 |
| Male ***X*** age 70–79 | 10.212 | [4.533, 23.008] | < 0.001 |
| Male ***X*** age 80+ | 2.281 | [0.819, 6.348] | 0.114 |
| **Other Communicable** |  |  |  |
| *Sex* |  |  |  |
| Male | 1.176 | [0.957, 1.445] | 0.122 |
| *10-Year Age Groups* |  |  |  |
| 0–4 | 1.000 | – | – |
| 5–9 | 0.055 | [0.029, 0.103] | < 0.001 |
| 10–19 | 0.051 | [0.031, 0.084] | < 0.001 |
| 20–29 | 0.128 | [0.087, 0.189] | < 0.001 |
| 30–39 | 0.192 | [0.131, 0.280] | < 0.001 |
| 40–49 | 0.192 | [0.122, 0.303] | < 0.001 |
| 50–59 | 0.146 | [0.077, 0.277] | < 0.001 |
| 60–69 | 0.177 | [0.093, 0.335] | < 0.001 |
| 70–79 | 0.705 | [0.466, 1.066] | 0.097 |
| 80+ | 1.519 | [0.921, 2.506] | 0.102 |
| *Time Period* |  |  |  |
| 1990–1994 | 1.000 | – | – |
| 1995–1999 | 1.213 | [0.899, 1.638] | 0.206 |
| 2000–2004 | 1.375 | [1.021, 1.853] | 0.036 |
| *Interactions between Sex and Age* |  |  |  |
| Male ***X*** age 5–9 | 0.677 | [0.261, 1.754] | 0.422 |
| Male ***X*** age 10–19 | 0.385 | [0.162, 0.915] | 0.031 |
| Male ***X*** age 20–29 | 0.555 | [0.304, 1.013] | 0.055 |
| Male ***X*** age 30–39 | 0.597 | [0.329, 1.082] | 0.089 |
| Male ***X*** age 40–49 | 1.054 | [0.560, 1.984] | 0.871 |
| Male ***X*** age 50–59 | 1.443 | [0.624, 3.334] | 0.391 |
| Male ***X*** age 60–69 | 1.989 | [0.861, 4.596] | 0.108 |
| Male ***X*** age 70–79 | 1.167 | [0.623, 2.186] | 0.629 |
| Male ***X*** age 80+ | 0.909 | [0.421, 1.963] | 0.809 |
| **Noncommunicable** |  |  |  |
| *Sex* |  |  |  |
| Male | 1.199 | [0.616, 2.331] | 0.594 |
| *10-Year Age Groups* |  |  |  |
| 0–4 | 1.000 | – | – |
| 5–9 | 0.289 | [0.106, 0.788] | 0.015 |
| 10–19 | 0.317 | [0.144, 0.699] | 0.004 |
| 20–29 | 1.215 | [0.654, 2.255] | 0.538 |
| 30–39 | 2.902 | [1.643, 5.128] | < 0.001 |
| 40–49 | 5.497 | [3.156, 9.573] | < 0.001 |
| 50–59 | 11.096 | [6.453, 19.079] | < 0.001 |
| 60–69 | 18.886 | [11.142, 32.012] | < 0.001 |
| 70–79 | 41.328 | [24.646, 69.303] | < 0.001 |
| 80+ | 86.192 | [50.604, 146.807] | < 0.001 |
| *Time Period* |  |  |  |
| 1990–1994 | 1.000 | – | – |
| 1995–1999 | 1.105 | [0.862, 1.418] | 0.43 |
| 2000–2004 | 1.341 | [1.050, 1.713] | 0.019 |
| *Interactions between Sex and Age* |  |  |  |
| Male ***X*** age 5–9 | 0.332 | [0.057, 1.950] | 0.222 |
| Male ***X*** age 10–19 | 1.044 | [0.362, 3.012] | 0.936 |
| Male ***X*** age 20–29 | 0.635 | [0.264, 1.525] | 0.31 |
| Male ***X*** age 30–39 | 0.529 | [0.233, 1.203] | 0.129 |
| Male ***X*** age 40–49 | 0.988 | [0.462, 2.112] | 0.975 |
| Male ***X*** age 50–59 | 1.362 | [0.655, 2.833] | 0.408 |
| Male ***X*** age 60–69 | 1.17 | [0.567, 2.418] | 0.671 |
| Male ***X*** age 70–79 | 1.433 | [0.707, 2.905] | 0.318 |
| Male ***X*** age 80+ | 1.101 | [0.530, 2.289] | 0.796 |
| **Injuries** |  |  |  |
| *Sex* |  |  |  |
| Male | 1.164 | [0.554, 2.447] | 0.689 |
| *10-Year Age Groups* |  |  |  |
| 0–4 | 1.000 | – | – |
| 5–9 | 0.57 | [0.236, 1.375] | 0.211 |
| 10–19 | 0.71 | [0.348, 1.450] | 0.348 |
| 20–29 | 1.063 | [0.525, 2.153] | 0.865 |
| 30–39 | 0.393 | [0.140, 1.102] | 0.076 |
| 40–49 | 2.047 | [0.994, 4.215] | 0.052 |
| 50–59 | 1.342 | [0.535, 3.365] | 0.53 |
| 60–69 | 1.619 | [0.646, 4.059] | 0.304 |
| 70–79 | 2.862 | [1.186, 6.908] | 0.019 |
| 80+ | 4.725 | [1.539, 14.506] | 0.007 |
| *Time Period* |  |  |  |
| 1990–1994 | 1.000 | – | – |
| 1995–1999 | 0.727 | [0.532, 0.992] | 0.044 |
| 2000–2004 | 0.846 | [0.624, 1.148] | 0.284 |
| *Interactions between Sex and Age* |  |  |  |
| Male ***X*** age 5–9 |  |  |  |
| Male ***X*** age 10–19 | 1.389 | [0.439, 4.398] | 0.576 |
| Male ***X*** age 20–29 | 1.931 | [0.765, 4.872] | 0.164 |
| Male ***X*** age 30–39 | 4.264 | [1.749, 10.395] | 0.001 |
| Male ***X*** age 40–49 | 16.803 | [5.225, 54.035] | < 0.001 |
| Male ***X*** age 50–59 | 2.984 | [1.184, 7.523] | 0.02 |
| Male ***X*** age 60–69 | 5.214 | [1.735, 15.668] | 0.003 |
| Male ***X*** age 70–79 | 4.324 | [1.392, 13.430] | 0.011 |
| Male ***X*** age 80+ | 3.403 | [1.108, 10.454] | 0.032 |

^a Multinomial logistic regression of adult death by cause on sex, age, and time period. Unit of analysis is “person-year.” Explanatory variables are defined at beginning of each year. Referent group is surviving adults.^
