# Supplementary material for: The Evolving Demographic and Health Transition in Four Low- and Middle-Income Countries: Evidence from Four Sites in the INDEPTH Network of Longitudinal Health and Demographic Surveillance Systems
Source: PLoS One. 2016 Jun 15;11(6):e0157281. doi: 10.1371/journal.pone.0157281 (PMC4909223; doi:10.1371/journal.pone.0157281)
Supplement: S7 Table — (DOCX) [file pone.0157281.s012.docx]

**Table S7. Multinomial logistic regression of cause-specific mortality, Navrongo, Ghana, 1995–2004 (N = 1,215,411 person years).**

| Variable | Odds Ratio | 95% CI | p-value |
| --- | --- | --- | --- |
| **Malaria** |  |  |  |
| *Sex* |  |  |  |
| Male | 0.976 | [0.897, 1.062] | 0.571 |
| *10-Year Age Groups* |  |  |  |
| 0–4 | 1.000 | – | – |
| 5–9 | 0.093 | [0.074, 0.118] | < 0.001 |
| 10–19 | 0.02 | [0.013, 0.030] | < 0.001 |
| 20–29 | 0.11 | [0.081, 0.149] | < 0.001 |
| 30–39 | 0.203 | [0.162, 0.253] | < 0.001 |
| 40–49 | 0.078 | [0.060, 0.100] | < 0.001 |
| 50–59 | 0.176 | [0.140, 0.222] | < 0.001 |
| 60–69 | 0.364 | [0.296, 0.448] | < 0.001 |
| 70–79 | 0.675 | [0.533, 0.855] | 0.001 |
| 80+ | 1.54 | [1.117, 2.123] | 0.008 |
| *Time Period* |  |  |  |
| 1995–1999 | 1.759 | [1.611, 1.921] | < 0.001 |
| 2000–2004 | 1.000 | – | – |
| *Interactions between Sex and Age* | |  |  |
| Male ***X*** age 5–9 | 1.359 | [1.028, 1.797] | 0.031 |
| Male ***X*** age 10–19 | 1.22 | [0.836, 1.781] | 0.302 |
| Male ***X*** age 20–29 | 0.561 | [0.378, 0.833] | 0.004 |
| Male ***X*** age 30–39 | 1.2 | [0.909, 1.584] | 0.198 |
| Male ***X*** age 40–49 | 1.762 | [1.327, 2.339] | < 0.001 |
| Male ***X*** age 50–59 | 2.075 | [1.627, 2.648] | < 0.001 |
| Male ***X*** age 60–69 | 1.331 | [1.062, 1.667] | 0.013 |
| Male ***X*** age 70–79 | 1.23 | [0.962, 1.573] | 0.099 |
| Male ***X*** age 80+ | 0.869 | [0.610, 1.237] | 0.435 |
| *Interactions between Age and Time* | |  |  |
| 1995–1999 ***X*** age 10–19 | 1.591 | [1.050, 2.411] | 0.028 |
| 1995–1999 ***X*** age 20–29 | 0.533 | [0.364, 0.779] | 0.001 |
| 1995–1999 ***X*** age 30–39 | 0.354 | [0.267, 0.469] | < 0.001 |
| 1995–1999 ***X*** age 50–59 | 0.709 | [0.555, 0.905] | 0.006 |
| 1995–1999 ***X*** age 60–69 | 0.921 | [0.733, 1.159] | 0.484 |
| 1995–1999 ***X*** age 70–79 | 1.156 | [0.898, 1.487] | 0.26 |
| 1995–1999 ***X*** age 80+ | 0.909 | [0.639, 1.294] | 0.596 |
| 2000–2004 ***X*** age 5–9 | 0.656 | [0.481, 0.895] | 0.008 |
| 2000–2004 X age 40–49 | 1.729 | [1.301, 2.298] | < 0.001 |
| **Other Communicable** |  |  |  |
| *Sex* |  |  |  |
| Male | 1.055 | [0.983, 1.132] | 0.136 |
| *10-Year Age Groups* |  |  |  |
| 0–4 | 1.000 | – | – |
| 5–9 | 0.077 | [0.062, 0.096] | < 0.001 |
| 10–19 | 0.062 | [0.050, 0.076] | < 0.001 |
| 20–29 | 0.142 | [0.116, 0.174] | < 0.001 |
| 30–39 | 0.142 | [0.117, 0.171] | < 0.001 |
| 40–49 | 0.211 | [0.182, 0.245] | < 0.001 |
| 50–59 | 0.416 | [0.366, 0.473] | < 0.001 |
| 60–69 | 0.944 | [0.842, 1.058] | 0.32 |
| 70–79 | 1.612 | [1.412, 1.841] | < 0.001 |
| 80+ | 3.319 | [2.772, 3.974] | < 0.001 |
| *Time Period* |  |  |  |
| 1995–1999 | 1.272 | [1.186, 1.366] | < 0.001 |
| 2000–2004 | 1.000 | – | – |
| *Interactions between Sex and Age* |  |  |  |
| Male X age 5–9 | 1.023 | [0.794, 1.318] | 0.861 |
| Male X age 10–19 | 0.957 | [0.762, 1.202] | 0.705 |
| Male X age 20–29 | 0.552 | [0.432, 0.705] | < 0.001 |
| Male X age 30–39 | 1.187 | [0.966, 1.457] | 0.103 |
| Male X age 40–49 | 1.805 | [1.517, 2.149] | < 0.001 |
| Male X age 50–59 | 1.243 | [1.083, 1.426] | 0.002 |
| Male X age 60–69 | 0.949 | [0.832, 1.081] | 0.428 |
| Male X age 70–79 | 0.905 | [0.781, 1.049] | 0.186 |
| Male X age 80+ | 0.826 | [0.674, 1.013] | 0.066 |
| *Interactions between Age and Time* |  |  |  |
| 1995–1999 X age 10–19 | 0.924 | [0.737, 1.160] | 0.498 |
| 1995–1999 X age 20–29 | 1.045 | [0.824, 1.326] | 0.714 |
| 1995–1999 X age 30–39 | 1.126 | [0.914, 1.389] | 0.266 |
| 1995–1999 X age 50–59 | 1.255 | [1.090, 1.444] | 0.002 |
| 1995–1999 X age 60–69 | 1.161 | [1.018, 1.323] | 0.026 |
| 1995–1999 X age 70–79 | 1.298 | [1.119, 1.507] | 0.001 |
| 1995–1999 X age 80+ | 1.034 | [0.844, 1.266] | 0.748 |
| 2000–2004 X age 5–9 | 0.987 | [0.764, 1.274] | 0.918 |
| 2000–2004 X age 40–49 | 0.821 | [0.688, 0.979] | 0.028 |
| **Noncommunicable** |  |  |  |
| *Sex* |  |  |  |
| Male | 1.956 | [0.734, 5.211] | 0.18 |
| *10-Year Age Groups* |  |  |  |
| 0–4 | 1.000 | – | – |
| 5–9 | 0.44 | [0.061, 3.192] | 0.417 |
| 10–19 | 4.978 | [2.035, 12.176] | < 0.001 |
| 20–29 | 10.994 | [4.520, 26.740] | < 0.001 |
| 30–39 | 17.999 | [7.519, 43.083] | < 0.001 |
| 40–49 | 65.118 | [21.551, 196.762] | < 0.001 |
| 50–59 | 67.257 | [28.541, 158.489] | < 0.001 |
| 60–69 | 147.634 | [62.775, 347.205] | < 0.001 |
| 70–79 | 270.608 | [114.775, 638.019] | < 0.001 |
| 80+ | 579.684 | [243.794, 1378.348] | < 0.001 |
| *Time Period* |  |  |  |
| 1995–1999 | 0.37 | [0.132, 1.038] | 0.059 |
| 2000–2004 | 1.000 | – | – |
| *Interactions between Sex and Age* |  |  |  |
| Male X age 5–9 | 0.772 | [0.136, 4.373] | 0.77 |
| Male X age 10–19 | 0.714 | [0.255, 1.998] | 0.522 |
| Male X age 20–29 | 0.812 | [0.292, 2.259] | 0.69 |
| Male X age 30–39 | 1.177 | [0.431, 3.216] | 0.751 |
| Male X age 40–49 | 1.092 | [0.403, 2.955] | 0.863 |
| Male X age 50–59 | 0.831 | [0.309, 2.234] | 0.713 |
| Male X age 60–69 | 0.693 | [0.258, 1.860] | 0.467 |
| Male X age 70–79 | 0.512 | [0.190, 1.379] | 0.185 |
| Male X age 80+ | 0.434 | [0.159, 1.182] | 0.103 |
| *Interactions between Age and Time* |  |  |  |
| 1995–1999 X age 10–19 | 1.997 | [0.681, 5.853] | 0.208 |
| 1995–1999 X age 20–29 | 1.983 | [0.679, 5.792] | 0.21 |
| 1995–1999 X age 30–39 | 1.748 | [0.609, 5.021] | 0.3 |
| 1995–1999 X age 50–59 | 1.982 | [0.700, 5.608] | 0.197 |
| 1995–1999 X age 60–69 | 2.132 | [0.755, 6.021] | 0.153 |
| 1995–1999 X age 70–79 | 2.454 | [0.866, 6.951] | 0.091 |
| 1995–1999 X age 80+ | 1.73 | [0.603, 4.965] | 0.309 |
| 2000–2004 X age 5–9 | 1.173 | [0.175, 7.875] | 0.869 |
| 2000–2004 X age 40–49 | 0.426 | [0.150, 1.214] | 0.11 |
| **Injuries** |  |  |  |
| *Sex* |  |  |  |
| Male | 0.948 | [0.744, 1.208] | 0.668 |
| *10-Year Age Groups* |  |  |  |
| 0–4 | 1.000 | – | – |
| 5–9 | 0.207 | [0.133, 0.321] | < 0.001 |
| 10–19 | 0.262 | [0.177, 0.390] | < 0.001 |
| 20–29 | 0.303 | [0.190, 0.486] | < 0.001 |
| 30–39 | 0.339 | [0.218, 0.528] | < 0.001 |
| 40–49 | 0.208 | [0.132, 0.328] | < 0.001 |
| 50–59 | 0.472 | [0.307, 0.725] | 0.001 |
| 60–69 | 0.758 | [0.490, 1.171] | 0.212 |
| 70–79 | 1.408 | [0.847, 2.340] | 0.187 |
| 80+ | 3.28 | [1.693, 6.355] | < 0.001 |
| *Time Period* |  |  |  |
| 1995–1999 | 1.416 | [1.106, 1.812] | 0.006 |
| 2000–2004 | 1.000 | – | – |
| *Interactions between Sex and Age* |  |  |  |
| Male X age 5–9 | 1.556 | [0.963, 2.516] | 0.071 |
| Male X age 10–19 | 1.777 | [1.167, 2.704] | 0.007 |
| Male X age 20–29 | 2.625 | [1.600, 4.305] | < 0.001 |
| Male X age 30–39 | 3.838 | [2.407, 6.119] | < 0.001 |
| Male X age 40–49 | 4.336 | [2.653, 7.085] | < 0.001 |
| Male X age 50–59 | 2.401 | [1.487, 3.878] | < 0.001 |
| Male X age 60–69 | 1.701 | [1.016, 2.849] | 0.043 |
| Male X age 70–79 | 1.067 | [0.570, 1.999] | 0.839 |
| Male X age 80+ | 0.316 | [0.125, 0.794] | 0.014 |
| *Interactions between Age and Time* |  |  |  |
| 1995–1999 X age 10–19 | 0.754 | [0.503, 1.130] | 0.171 |
| 1995–1999 X age 20–29 | 0.714 | [0.450, 1.134] | 0.154 |
| 1995–1999 X age 30–39 | 0.635 | [0.410, 0.984] | 0.042 |
| 1995–1999 X age 50–59 | 0.538 | [0.333, 0.868] | 0.011 |
| 1995–1999 X age 60–69 | 0.465 | [0.273, 0.791] | 0.005 |
| 1995–1999 X age 70–79 | 0.479 | [0.248, 0.922] | 0.028 |
| 1995–1999 X age 80+ | 0.917 | [0.389, 2.159] | 0.842 |
| 2000–2004 X age 5–9 | 1.734 | [1.081, 2.783] | 0.023 |
| 2000–2004 X age 40–49 | 1.309 | [0.832, 2.062] | 0.244 |

^a Multinomial logistic regression of adult death by cause on sex, age, and time period. Unit of analysis is “person-year.” Explanatory variables are defined at beginning of each year. Referent group is surviving adults.^
