# Supplementary material for: The Evolving Demographic and Health Transition in Four Low- and Middle-Income Countries: Evidence from Four Sites in the INDEPTH Network of Longitudinal Health and Demographic Surveillance Systems
Source: PLoS One. 2016 Jun 15;11(6):e0157281. doi: 10.1371/journal.pone.0157281 (PMC4909223; doi:10.1371/journal.pone.0157281)
Supplement: S8 Table — (DOCX) [file pone.0157281.s013.docx]

**Table S8. Multinomial logistic regression of cause-specific mortality, Filabavi, Vietnam, 2004–2007 (N = 144,713 person years).**

| Variable | Odds Ratio | 95% CI | p-value |
| --- | --- | --- | --- |
| **Communicable** |  |  |  |
| *Sex* |  |  |  |
| Male | 1.738 | [1.268, 2.382] | 0.001 |
| *10-Year Age Groups* |  |  |  |
| 0–4 | 1.000 | – | – |
| 5–9 | 0.096 | [0.012, 0.739] | 0.024 |
| 10–19 | 0.109 | [0.031, 0.386] | 0.001 |
| 20–29 | 0.146 | [0.041, 0.518] | 0.003 |
| 30–39 | 0.443 | [0.181, 1.083] | 0.074 |
| 40–49 | 0.2 | [0.064, 0.620] | 0.005 |
| 50–59 | 0.273 | [0.077, 0.967] | 0.044 |
| 60–69 | 1.337 | [0.563, 3.175] | 0.511 |
| 70–79 | 4.959 | [2.505, 9.817] | < 0.001 |
| 80+ | 39.468 | [21.477, 72.529] | < 0.001 |
| **Noncommunicable** |  |  |  |
| *Sex* |  |  |  |
| Male | 2.273 | [1.892, 2.730] | < 0.001 |
| *10-Year Age Groups* |  |  |  |
| 0–4 | 1.000 | – | – |
| 5–9 | 0.289 | [0.061, 1.363] | 0.117 |
| 10–19 | 0.164 | [0.043, 0.617] | 0.008 |
| 20–29 | 0.811 | [0.326, 2.017] | 0.653 |
| 30–39 | 1.594 | [0.698, 3.643] | 0.269 |
| 40–49 | 4.321 | [2.061, 9.060] | < 0.001 |
| 50–59 | 7.068 | [3.352, 14.902] | < 0.001 |
| 60–69 | 15.011 | [7.201, 31.290] | < 0.001 |
| 70–79 | 43.224 | [21.196, 88.147] | < 0.001 |
| 80+ | 84.978 | [41.477, 174.103] | < 0.001 |
| **Injuries** |  |  |  |
| *Sex* |  |  |  |
| Male | 3.154 | [2.049, 4.853] | < 0.001 |
| *10-Year Age Groups* |  |  |  |
| 0–4 | 1.000 | – | – |
| 5–9 | 0.387 | [0.040, 3.726] | 0.412 |
| 10–19 | 1.898 | [0.541, 6.663] | 0.317 |
| 20–29 | 4.568 | [1.371, 15.219] | 0.013 |
| 30–39 | 3.623 | [1.055, 12.438] | 0.041 |
| 40–49 | 2.455 | [0.692, 8.701] | 0.164 |
| 50–59 | 0.752 | [0.126, 4.504] | 0.755 |
| 60–69 | 0.62 | [0.065, 5.966] | 0.679 |
| 70–79 | 6.465 | [1.712, 24.416] | 0.006 |
| 80+ | 43.558 | [12.983, 146.143] | < 0.001 |

^a Multinomial logistic regression of adult death by cause on sex, age, and time period. Unit of analysis is “person-year.” Explanatory variables are defined at beginning of each year. Referent group is surviving adults.^
