# Supplementary material for: The Evolving Demographic and Health Transition in Four Low- and Middle-Income Countries: Evidence from Four Sites in the INDEPTH Network of Longitudinal Health and Demographic Surveillance Systems
Source: PLoS One. 2016 Jun 15;11(6):e0157281. doi: 10.1371/journal.pone.0157281 (PMC4909223; doi:10.1371/journal.pone.0157281)
Supplement: S9 Table — (DOCX) [file pone.0157281.s014.docx]

**Table S9: Multinomial logistic model of all-cause mortality on cause-specific mortality at the individual level, by DSS site.**

|  | Matlab | | | Agincourt | | | Navrongo | | |
| --- | --- | --- | --- | --- | --- | --- | --- | --- | --- |
|  | Odds ratio | 95% CI | p-value | Odds ratio | 95% CI | p-value | Odds ratio | 95% CI | p-value |
| Communicable |  |  |  |  |  |  |  |  |  |
| All-cause mortality | 9.155 | [8.053, 10.408] | < 0.001 | 0.845 | [0.759, 0.941] | 0.002 | 121.314 | [102.668, 143.346] | < 0.001 |
| Noncommunicable |  |  |  |  |  |  |  |  |  |
| All-cause mortality | 0.105 | [0.092, 0.120] | < 0.001 | 0.504 | [0.434, 0.584] | < 0.001 | 14.652 | [11.914, 18.019] | < 0.001 |
| Injuries |  |  |  |  |  |  |  |  |  |
| All-cause mortality | 1.097 | [0.836, 1.441] | 0.503 | 0.383 | [0.305, 0.481] | < 0.001 | 30.187 | [18.869, 48.296] | < 0.001 |
