# Supplementary material for: The Evolving Demographic and Health Transition in Four Low- and Middle-Income Countries: Evidence from Four Sites in the INDEPTH Network of Longitudinal Health and Demographic Surveillance Systems
Source: PLoS One. 2016 Jun 15;11(6):e0157281. doi: 10.1371/journal.pone.0157281 (PMC4909223; doi:10.1371/journal.pone.0157281)
Supplement: S10 Table — (DOCX) [file pone.0157281.s015.docx]

**Table S10: Seemingly unrelated regression model of all-cause mortality on cause-specific mortality at the site-level.**

|  | Beta | 95% CI | p-value |
| --- | --- | --- | --- |
| **Noncommunicable** |  |  |  |
| Log all-cause mortality | -1.682 | [-1.959, -1.404] | < 0.001 |
| Agincourt | -0.241 | [-0.390, -0.092] | 0.002 |
| Navrongo | 0.848 | [0.473, 1.222] | < 0.001 |
| 1988 | 0.466 | [0.064, 0.868] | 0.023 |
| 1989 | 0.839 | [0.437, 1.241] | < 0.001 |
| 1990 | 0.571 | [0.168, 0.973] | 0.005 |
| 1991 | 0.603 | [0.200, 1.006] | 0.003 |
| 1992 | 0.833 | [0.430, 1.237] | < 0.001 |
| 1993 | 0.723 | [0.319, 1.126] | < 0.001 |
| 1994 | 0.957 | [0.587, 1.326] | < 0.001 |
| 1995 | 0.904 | [0.555, 1.254] | < 0.001 |
| 1996 | 0.885 | [0.534, 1.235] | < 0.001 |
| 1997 | 0.868 | [0.518, 1.218] | < 0.001 |
| 1998 | 0.93 | [0.580, 1.280] | < 0.001 |
| 1999 | 1.009 | [0.660, 1.359] | < 0.001 |
| 2000 | 1.34 | [0.999, 1.681] | < 0.001 |
| 2001 | 1.425 | [1.085, 1.766] | < 0.001 |
| 2002 | 1.483 | [1.143, 1.824] | < 0.001 |
| 2003 | 1.511 | [1.171, 1.851] | < 0.001 |
| 2004 | 1.741 | [1.401, 2.080] | < 0.001 |
| 2005 | 1.714 | [1.291, 2.137] | < 0.001 |
| Constant | 0.741 | [0.280, 1.202] | 0.002 |
| **Injuries** |  |  |  |
| Log all-cause mortality | -1.477 | [-1.814, -1.139] | < 0.001 |
| Agincourt | 0.38 | [0.199, 0.562] | < 0.001 |
| Navrongo | 0.731 | [0.275, 1.186] | 0.002 |
| 1988 | 0.165 | [-0.324, 0.654] | 0.507 |
| 1989 | 0.136 | [-0.353, 0.625] | 0.584 |
| 1990 | -0.084 | [-0.574, 0.406] | 0.737 |
| 1991 | -0.264 | [-0.754, 0.226] | 0.29 |
| 1992 | -0.262 | [-0.752, 0.229] | 0.296 |
| 1993 | -0.03 | [-0.521, 0.460] | 0.904 |
| 1994 | 0.312 | [-0.137, 0.761] | 0.173 |
| 1995 | 0.164 | [-0.261, 0.589] | 0.449 |
| 1996 | -0.082 | [-0.509, 0.344] | 0.705 |
| 1997 | 0.006 | [-0.420, 0.432] | 0.979 |
| 1998 | 0.092 | [-0.334, 0.517] | 0.673 |
| 1999 | 0.067 | [-0.358, 0.492] | 0.757 |
| 2000 | 0.085 | [-0.329, 0.499] | 0.687 |
| 2001 | 0.308 | [-0.107, 0.722] | 0.146 |
| 2002 | 0.327 | [-0.087, 0.740] | 0.122 |
| 2003 | 0.259 | [-0.154, 0.673] | 0.219 |
| 2004 | 0.621 | [0.209, 1.034] | 0.003 |
| 2005 | 0.063 | [-0.451, 0.578] | 0.809 |
| Constant | 0.015 | [-0.545, 0.575] | 0.958 |
